# Four types of adenine-related RNA modification writers -mediated molecular subtypes contribute to predicting clinical outcomes and treatment options in bladder cancer

**DOI:** 10.3389/fimmu.2023.1152806

**Published:** 2023-08-11

**Authors:** Yao Zhang, Ying Chen, Wen Wen

**Affiliations:** ^1^ Department of gynaecology, Shengjing Hospital of China Medical University, Shenyang, China; ^2^ Department of Ultrasound, Xiaoshan Traditional Chinese Medical Hospital, Hangzhou, China; ^3^ Department of Laboratory Medicine, Shengjing Hospital of China Medical University, Liaoning Clinical Research Center for Laboratory Medicine, Shenyang, China

**Keywords:** RNA modification writers, bladder cancer, immunotherapy benefits, tumor microenvironment, unsupervised clustering

## Abstract

**Rationale:**

RNA modifications, containing m6A, m1A, alternative polyadenylation and adenosine-to-inosine RNA editing, involve in critical cancerous immunity and cancerous processes. However, the functional roles of RNA modification writers in bladder cancer (BLCA) are largely unknown.

**Methods:**

In this study, unsupervised clustering was used to identify novel RNA modification writers -mediated molecular subtypes in BLCA. A corresponding quantitative indicator called WriterScore was developed using univariate Cox and Least absolute shrinkage and selection operator (LASSO) analysis. Then, we systematically analyzed the correlation between RNA modification writer-related clusters (WriterScore) and immunological characteristics, classical molecular subtypes, clinicopathologic features and treatment options in BLCA. Finally, we validated the WriterScore in multiple other external BLCA datasets, clinical sample dataset in Shengjing Hospital and pancancer.

**Results:**

Two RNA modification writer-related clusters and three DEGclusters were obtained. These RNA modification writer-related clusters (WriterScore) were strongly associated with immunological characteristics, classical molecular subtypes, clinicopathologic features of BLCA. Moreover, WriterScore can properly predict the clinical outcomes and immunotherapy of BLCA patients.

**Conclusion:**

Our study systematically investigated the role of RNA modification writers and developed a significant WriterScore to guide several treatment options in BLCA, which might bring some potential benefits for BLCA patients.

## Background

Bladder cancer (BLCA) is the second most prevalent malignancies in the urinary system (1), with an estimated 81,180 new cases and about 17,100 deaths worldwide in the United States in 2022 (2). The main therapeutic methods include surgery, radiotherapy, chemotherapy, immunotherapy, etc. However, the prognosis for BLCA patients remains unsatisfactory (3, 4). The main reason is that most BLCA patients are not sensitive to these treatment strategies, and there are no robust tools and biomarkers to accurately predict clinical response to these treatment protocols. Therefore, it is urgent to develop more effective biomarkers and methods to predict the treatment benefits for BLCA patients.

Epigenetic alterations modulating heritable changes play important role in the malignant process of human cancer (5). RNA modification, as an essential part in epigenetic alterations, play a role in many pathological processes of cancer. More than 170 different types of RNA modifications have been described to modify coding and non-coding RNAs so far (6). Adenine is the most modified nucleotide on RNA and N6-methyladenosine (m6A) is the most abundant internal modification of mRNA (7, 8). Except for the most common m6A methylation, N1-methyladenosine (m1A), alternative polyadenylation (APA) and ADAR-induced adenine to inosine (A-to-I) are also adenine-related RNA modifications. The m6A modification can regulate the processes of mRNA stability, mRNA export, transcription, translation and pre-mRNA splicing (9–11). APA has been reported in over 60% of human genes, and affect various gene regulation processes, such as cellular RNA decay, mRNA stability, mRNA maturation, and protein diversification (12). The m1A occurs widely in rRNA, tRNA, mRNA and mitochondrial transcripts (13). In mRNA, it usually distributed in the 5’-UTR of mRNA and participate in translation (14). In addition, it also has been reported delay reverse transcription and participate in the regulation of cellular stress response (15). A-to-I RNA editing has been found in miRNAs, lncRNAs, pre-mRNAs, tRNAs and mRNAs. It can regulate co-transcriptional and post-transcriptional modification though converting adenosines to inosines (16). RNA modifications were regulated by multiple regulatory proteins encoded by ‘writer’ (installer), ‘reader’ (translator) and ‘eraser’ (demodifier) (17). Write proteins transfer specific chemical groups to target sites on RNA molecules; Reader proteins specifically recognize modified nucleotides; Eraser proteins remove specific chemical groups from modified nucleotides and convert them back to unmodified nucleotides (17). In the present study, we systematically investigated the functional role of adenine-related RNA modification writers in BLCA.

In the past decade, immunotherapies have been conducted great progress and shown tremendous assistant to advanced solid tumors. In 2016, immune checkpoint inhibitors (ICIs) have been formally applied in BLCA patients (18), and have achieved certain therapeutic effects due to the strong immunogenicity (19). Although immunotherapies for BLCA have long been proved to be safe and effective, the response rate is still less than 30% (20, 21). Thus, it is worthwhile to explore the potential mechanisms of BLCA in tumor microenvironment (TME). Previous studies have reported that TME has close correlation with adenine-related RNA modifications. For example, Xueqing Hu et al. have demonstrated that YAP1 may promote BLCA progression through suppressing the CD4+ Th1 cells, T follicular helper cells, NKT cells, infiltration of CD8+ T lymphocytes and activated NK cells (22). Yuzhen Gao et al. have reported that m1A regulators mediated three distinct immunophenotype (desert, excluded and inflamed) of in TME -infiltrating immune cells in colon cancer (23). However, the interaction of these four adenine-related RNA modification writers with TME in BLCA remains unclear. Thus, it is significant to systematically reveal biological mechanism by which four adenine-related RNA modification writers involving in TME of BLCA, as this may be a promising method for achieving precision treatment in BLCA.

Four types of adenine modifications, including m6A, m1A, APA and A-to-I RNA editing, are the most impactful RNA modifications. In this study, we comprehensively evaluated the correlation of four types of adenine-related RNA modification writers with immunological characteristics, classical molecular subtypes, therapeutic opportunities, clinicopathological features for BLCA. Next, we developed two RNA modification writer-related clusters and three DEGclusters based on unsupervised clustering. Finally, we established and verified a WriterScore to quantify the efficacy of RNA modifications patterns in individual BLCA patients and evaluate its application value in predicting immunotherapy benefits.

## Materials and methods

### Tissue collection

Sixty BLCA samples and twenty normal samples were obtained from Tissue specimen Bank of Shengjing Hospital from 2015 to 2022. None of the patients in this study received preoperative radiotherapy or chemotherapy. The baseline information of the BLCA samples were presented in **Table S1C**. This study was approved by the Ethics Committee of Shengjing Hospital of the China Medical University, and informed consent was obtained from all patients. In addition, all methods were performed in accordance with relevant guidelines and regulations.

### RNA sequencing

Total RNA of the BLCA samples was extracted by TRIzol (Invitrogen, USA). The purity and concentration of the RNA samples were measured by NanoDropND-1000 (Thermo Fisher Scientific, USA). Subsequently, we removed rRNA from total RNA and obtained mRNA. Each sample was amplified and transcribed into a fluorescent cRNA using a random primer method. The sample RNA was first generated into cDNA by reverse transcription, and then purified and labeled. Finally, Agilent Gene Expression Hybridization Kit was used to hybridize the labeled probe and chip under standard conditions. We obtained the chip map and read the values to get the original data using Agilent Feature Extraction software. Then, GeneSpring GX v12.1 software (Agilent Technologies) was used to process the original data.

### Quantitative real-time RT-PCR

Total RNA from normal samples and BLCA samples was extracted by TRIzol (Invitrogen, USA) and reverse-transcribed to cDNA. Real time quantitative polymerase chain reaction (RT-qPCR) was performed based on SYBR Premix Exaq (Takara, Japan). GAPDH was used as an internal reference to calculate the relative expression levels of 26 RNA modification writers according to the 2-ΔΔCt method. **Supplementary Table 11** presents the primer sequences of the 26 RNA modification writers. We then compared the differential expression level of genes between Sixty BLCA samples and thirty normal samples.

### Data acquisition

The RNA sequencing data, somatic mutation data, Copy Number Variation (CNV) data and clinical data of TCGA- BLCA and pan-cancers were downloaded from the UCSC Xena Public Hub (http://xena.ucsc.edu/). As a training dataset in this study, TCGA- BLCA dataset included 400 BLCA samples and 19 normal samples after filtering the patients without prognostic information. Then, the FPKM values of the RNA sequencing data were transformed into transcripts per kilobase million (TPM) value. The “maftools”R package was used to plot the “oncoplot” based on the somatic mutation data. Tumor mutation burden (TMB) of pan-cancers was calculated using VarScan2 based on the mutation data. The microsatellite instability (MSI) data and the stemness indices of pan-cancers were respectively downloaded from the supplementary files of Bonneville’s study (24) and Malta’s study (25).

GSE48075 and GSE32894 from the same microarray platform (GPL6947 Illumina HumanHT-12 V3.0 expression beadchip) were downloaded from Gene Expression Omnibus (GEO, https://www.ncbi.nlm.nih.gov/). We then combined and normalized the two datasets into a meta-GEO dataset using the “sva” R package and the “gcrma” R package. The RNA sequencing data and clinical data of E-MTAB-4321 dataset was downloaded from http://E-MTAB-4321<ArrayExpress<BioStudies<EMBL-EBI. Subsequently, nine immunotherapy cohorts including IMvigor210 (BLCA, http://research-pub.Gene.com/imvigor210corebiologies/), GSE135222 (NSCLC, GEO), GSE78220 (Melanoma, GEO), GSE100797 (Melanoma, GEO), Gide2019 (Melanoma, TIDE website, http://tide.dfci.harvard.edu/download/), Nathanson2017_ Post and _Pre (Melanoma, TIDE website), Riaz2017_ Naïve and Prog (Melanoma, TIDE website) were obtained. Finally, a public BLCA single-cell data set (GSE145137) was downloaded from GEO dataset to explore the expression profiles of RNA modification writers on the cell clusters and cell types using “Seurat” R package. The microarray datasets from GEO were directly downloaded the present log scale matrix files. The baseline information about all of these datasets is collected in **Tables S1A–P**.

### Unsupervised clustering of RNA modification writers

Unsupervised clustering analysis was performed to detect novel RNA modification writers -mediated molecular subtypes in BLCA using the “ConsensuClusterPlus” R package based on the expression profiles of 26 RNA modification writers: 3 A-to-I enzymes (ADARB1, ADARB2 and ADAR), 4 m1A enzymes (TRMT6, TRMT61A, TRMT61B and TRMT10C), 7 m6A enzymes (RBM15, RBM15B, METTL3, METTL14, WTAP, KIAA1429 and ZC3H13) and 12 APA enzymes (CLP1, CPSF1/2/3/4, CFI, CSTF1/2/3, PABPN1, NUDT21 and PCF11) (**Table S2A**). Thousand resamplings were performed to maintain the stability of the clusters (26).

### Construction and validation of a Writer-score

We identified the RNA modification writer subtypes -related DEGs using the “Limma” R package based on the screening criteria P value < 0.05 and |logFC| > 20. Gene Ontology (GO) and Kyoto Encyclopedia of Genes and Genomes (KEGG) analyses were performed to explore the functions of DEGs in BLCA using the “clusterProfiler” R package. Univariate Cox regression was conducted to select the DEGs with prognostic value for further analysis. Unsupervised clustering was performed to classify TCGA- BLCA patients into different geneclusters based on the expression level of the prognostic-related DEGs to verify the stability of the RNA modification writers related phenotypes. Next, the LASSO regression analysis was performed to establish the Writer-score scoring system to quantify all individuals with BLCA based on the following formula: Writer-Score = ∑(Coefi * Expri); here, i means the genes in LASSO model, Coefi respects the coefficient of each gene and Expri indicates the expression level of each gene. The patients were classified into high-and low- Writer-Score groups using the “survminer” R package. The robustness of Writer-score scoring system was verified using meta-GEO dataset, E-MTAB-4321 dataset and clinical sample dataset.

### The association of Writer-score and classical molecular subtypes, classical molecular subtype-specific signatures

The information of several classical molecular subtypes including CIT, MDA, Lund, TCGA, UNC, Baylor and Consensus subtypes was collected from predecessors’ research (**Table S2B**) (27–33). Twelve classical molecular subtype-specific signatures were collected from The Bladder Cancer Molecular Taxonomy Group, such as Urothelial_differentiation, Ta_pathway, Luminal_differentiation, Basal_differentiation, EMT_differentiation, Immune_differentiation, Smooth_muscle, Myofibroblasts, Myofibroblasts, Interferon_response, Mitochondria, Keratinization and Neuroendocrine_differentiation (**Table S2C**) (33). The enrichment scores of 12 signatures were calculated using the “GSVA” R package. Finally, we correlated Writer-score with 7 molecular subtypes and 12 classical molecular subtype-specific signatures.

### The association of Writer-score and immunological characteristics

Immunological characteristics involved in BLCA were composed of 122 immunomodulators, tumor-infiltrating immune cells (TIICs), 36 effector genes of TIICs, cancer immunity cycles, several stromal signatures, T cell inflamed score (TIS) and 22 inhibitory immune checkpoints (**Tables S2D–I**) (34) (35, 36) (37). The infiltration level of TIICs in BLCA samples were calculated using the following algorithms: Cibersort-ABS, Cibersort, MCP-counter, TIMER, quanTIseq, TIP and xCell (**Table S2J**) (38–43). The cancer immunity cycle contains seven steps: Step 1.release of cancer cell antigens, Step 2.cancer antigen presentation, Step 3.priming and activation, Step 4.trafficking of immune cells to tumors (B.cell.recruiting, Basophil.recruiting, CD4.T.cell.recruiting, CD8.T.cell.recruiting, Dendritic.cell.recruiting, Eosinophil.recruiting, Macrophage.recruiting, MDSC.recruiting, Monocyte.recruiting, Neutrophil.recruiting, NK.cell.recruiting, T.cell.recruiting, Th1.cell.recruiting, Th17.cell.recruiting, Th2.cell.recruiting, Th22.cell.recruiting and Treg.cell.recruiting), Step 5.infiltration of immune cells into tumors, Step 6.recognition of cancer cells by T cells, and Step 7.killing of cancer cells (44). The activities of these steps were calculated using a single sample gene set enrichment analysis (ssGSEA) (43). The pan-cancer TIS, which could reflect the clinical response of Immune checkpoint blockade (ICB), was calculated using the T cell-inflamed score algorithm: TIS=∑(Coefi * Expri), here, i means the 18 genes (CCL5, CD27, CD274, CD276, CD8A, CMKLR1, CXCL9, CXCR6, HLA-DQA1, HLA-DRB1, HLA-E, IDO1, LAG3, NKG7, PDCD1LG2, PSMB10, STAT1 and TIGIT), Coefi respects the coefficient of each gene and Expri indicates the expression level of each gene (**Table S2H**) (45).

### The association of Writer-score and therapeutic-specific signatures

Several gene signatures correlated with the clinical response to an anti-PD-L1 agent (atezolizumab) in BLCA were summarized from Mariathasan’s study (**Table S2K**) (36). A set of therapeutic signatures, such as chemotherapies targeting immune-inhibited oncogenic pathways, EGFR targeted therapies and radiotherapies, were collected from Hu J’s study (**Table S2L**) (46). The enrichment scores of these signatures were calculated using the “GSVA” R package. Additionally, we also collected some predictors (RB1, ATM, ERBB2, ERCC2, and FANCC), the mutation status of which can reflect the BLCA patients’ sensitivity to neoadjuvant chemotherapy (47). Finally, we assessed the predictive value of the RNA modification writer-related clusters (WriterScore) to these therapies.

### Gene set variation analysis (GSVA) and functional annotation

GSVA enrichment analysis was conducted using “GSVA” R package to explore the differential biological function between distinct RNA modification writer-related clusters (WriterScore) (48). We downloaded the gene set c2.cp.kegg.v7.4.symbols.gmt, h.all.v2022.1.Hs.symbols.gmt and c6.all.v2022.1.Hs.symbols.gmt from the MSigDB database (https://www.gsea-msigdb.org/gsea/msigdb). False discovery rate (FDR) was corrected by Benjamini and Hochberg (BH) method and FDR < 0.05 was considered as signatures.

### Statistical analysis

All statistical data analyses were conducted using R software (version 4.1.2). The level of significance was set at P < 0.05, and all statistical tests were two-sided. Correlation analysis was applied using Pearson or Spearman coefficients. T-test or the Mann-Whitney U test was performed to the comparison among groups. Chi-square and Fisher’s exact tests were used to ensure exact test. Kaplan-Meier (K-M) survival curves was plotted to assess prognostic value and the statistical significance was evaluated by log-rank test.

## Results

### Landscape of 26 RNA modification writers in TCGA- BLCA dataset

**Figure 1:**
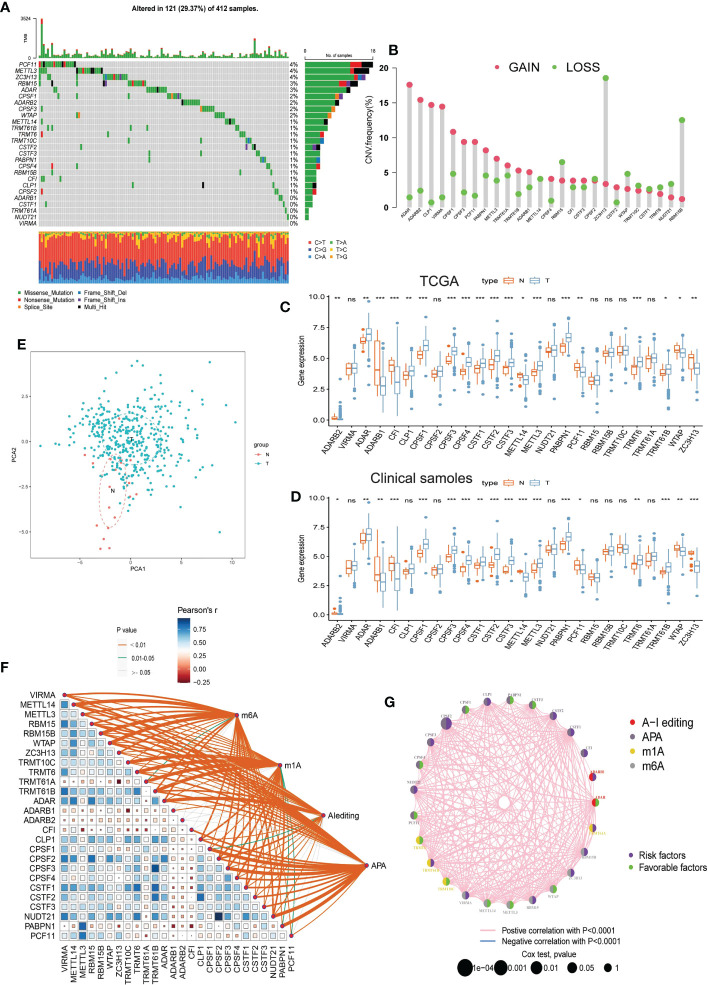
Landscape of 26 RNA modification writers in TCGA- BLCA dataset. **(A)** The mutation profiles of 26 RNA modification writers in 412 samples. **(B)** The copy number variation frequency of 26 RNA modification writers. **(C)** Differential expression histogram of the 26 RNA modification writers between BLCA and normal samples in TCGA- BLCA dataset. **(D)** Differential expression histogram of the 26 RNA modification writers between BLCA and normal samples in clinical sample dataset. **(E)** PCA plot of the BLCA and normal samples based on 26 RNA modification writers. **(F)** The correlations between 26 RNA modification writers. **(G)** The interactions between 26 RNA modification writers and their prognostic value for BLCA. The circle size indicates the p value of the log-rank test, and the lines linking the 26 RNA modification writers indicate their interactions. TCGA, The Cancer Genome Atlas; BLCA, bladder cancer; CNV, copy number variant; PCA, Principal Component Analysis; N, normal samples; T, tumor samples. *p<0.05, **p<0.01, *** P<0.001, ns:p<o,05.

26 RNA modification writers were collected in the current study based on the recent studies (49, 50). In the TCGA-BLCA dataset, the somatic mutation frequency of 26 RNA modification writers were not very frequent in BLCA. Of the 412 BLCA samples, 121 (29.37%) had mutations of RNA modification writers (**Figure 1A**). Among them, the mutation frequency of PCF11, METTL3, ZC3H13 was the highest (4%). We then assessed the CNV alterations of RNA modification writers, and found that ADAR, ADARB2, CLP1 and VRMA had high frequency of CNV gain, while ZC3H13 and RBM15B had a high proportion of CNV loss (**Figure 1B**). **Figures 1C, D** showed that RNA modification writers with CNV gain have relatively higher expression levels in tumor samples compared the normal samples, suggesting that CNV may be an essential factor to writers’ expression. Moreover, we used the single-cell RNA sequence dataset (GSE145137) to verify the overexpression patterns of RNA modification writers on BLCA epithelial cells (**Figure S1**). Qualified single cells were distributed into T cells, Epothelial cells, Monocyte, Endothelial cells, Fibrobiasts and Tissue stem cells. A majority of RNA modification writers, such as ADAR, CLP1, CPSF1/2/3/4, CSTF1/2/3, METTL3, PABPN1, TRMT6 and TRMT61B, were overexpressed on BLCA epithelial cells. Principal component analysis (PCA) showed that the 26 RNA modification writers can effectively separate BLCA samples from normal samples (**Figure 1E**). Pearson correlation analysis revealed that the majority of writers have positive correlation with each other (**Figures 1F, G**). K-M curve revealed that ADARB1, CFI, CPSF2, CPSF3, CSTF1, CSTF2, TRMT61A, VIRMA and ZC3H13 were poor prognostic factors, while ADARB2, CPSF1, CPSF4, CSTF3, METTL14, PABPN1, PCF11 and WTAP were protective prognostic factors (**Figure S2**). The above results demonstrated that RNA modification writers were potential predictors for BLCA diagnosis and prognosis.

### Unsupervised clustering based on 26 RNA modification writers

The patients in TCGA- BLCA dataset were divided into several clusters based on the expression of 26 RNA modification writers using unsupervised clustering. We found that the clustering algorithm achieve optimal result when the patients were classified into two clusters, including clusterA (n = 122) and clusterB (n = 284), and the patients in the two clusters have different prognosis (**Figures S3A–H**). The majority of 26 RNA modification writers were higher expressed in clusterA (**Figures S3I, K**). PCA analysis showed that the 26 RNA modification writers can effectively separate clusterA and clusterB group (**Figure S3L**). GSVA enrichment analysis was performed to investigate the biological function in different RNA modification patterns. ClusterB was markedly enriched in proliferation and apoptosis pathways, such as cell cycle, nucleotide excision repair, and mismatch repair pathways. ClusterA was mainly enriched in metabolism-related pathway, including metabolism of xenobiotics by cytochrome p450, drug metabolism cytochrome p450 and linoleic acid metabolism (**Figure S3J**; **Table S3**).

### Developing the Writer-score scoring system

**Figure 2:**
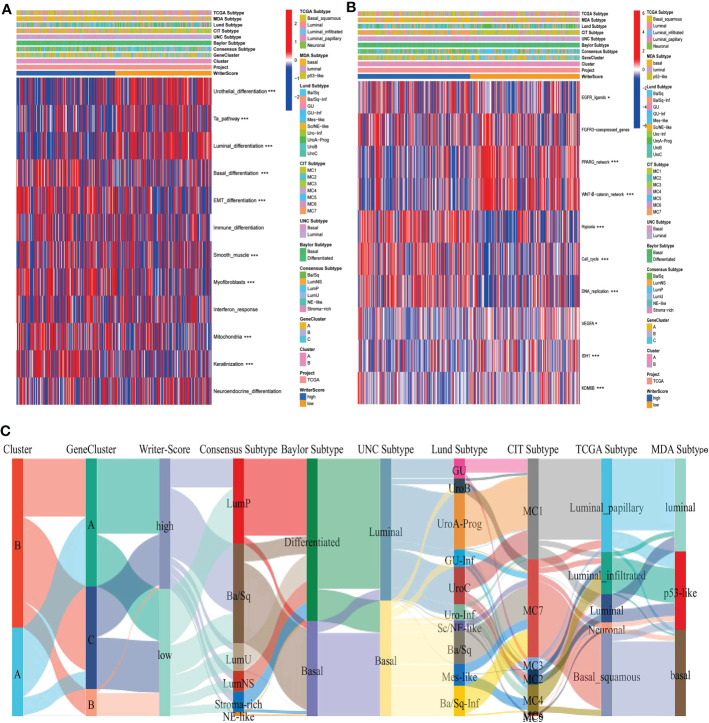
RNA modification patterns accurately predicted classical molecular subtypes and therapeutic opportunities in the TCGA-BLCA dataset. **(A, C)** The correlations between Clusters, geneClusters, Writer-Score, and seven classical molecular subtype classifications. **(B)** The correlations between the Writer-Score and the enrichment scores of several therapeutic signatures, such as EGFR targeted therapy, radiotherapy, chemotherapies targeting immune-inhibited oncogenic pathways. TCGA, The Cancer Genome Atlas; BLCA, bladder cancer. *p<0.05, *** P<0.001.

Among the two clusters, we subsequently identified 1525 RNA modification-related DEGs (**Figures S4A, B**; **Table S4A**), and enrichment analysis was performed based on the DEGs. GO enrichment analysis indicated that these genes were mainly associated to RNA transcription and translation processes (**Figure S4C**; **Table S4B**). KEGG enrichment analysis showed that were mainly enriched in proliferation and apoptosis pathways (**Figure S4D**; **Table S4C**). Next, 355 DEGs with significant prognostic value were selected (**Table S4D**). To further validate the different RNA modification patterns in TCGA-BLCA, unsupervised clustering was applied based on the expression of the 355 DEGs. We found that the patients were classified into three genomic phenotypes: geneClusterA, geneClusterB and geneClusterC, and the patients in geneClusterC had a poorer prognosis than patients in the other groups (**Figures S5A–H**). **Figure S5I** indicated that the majority of the 355 DEGs were higher expressed in geneClusterA group. Multivariable Cox analysis revealed that the RNA modification patterns was an independent prognostic factor for BLCA (**Figure S5K**; **Table S10A**). Subsequently, we developed a Writer-Score scoring system to quantify the RNA modification patterns. Firstly, we put the 355 DEGs into LASSO regression analysis and 18 genes were obtained (**Figures S4E, 4F**). Then, we put the 18 genes into the multivariate Cox regression analysis and achieved 10 genes to establish the Writer-Score scoring system. The coefficients of the 10 genes in the Writer-Score scoring system were used to calculate the Writer-Score of each patient (**Table S4E**). The patients in TCGA-BLCA dataset were divided in to high- or low writer-score groups according to an optimal cutoff value of the Writer-Score, and patients in high writer-score group have poor prognosis than the patients in low writer-score group (**Figure S4G**). The majority of the 26 RNA modification writers were higher expressed in low writer-score group (**Figure S5J**). The relationships among Cluster, geneCluster and Writer-Score were presented in **Figures S4H–J**. We found that the patients in ClusterB and geneClusterA, geneClusterC have higher Writer-Score. Furthermore, the hallmark pathways, KEGG pathways, oncogenic pathways and mutation profiles between high- and low writer-score group were compared using GSVA analysis. A majority of hallmark pathways such as MTORC1 signaling, hypoxia, epithelial mesenchymal transition and angiogenesis, oncogenic pathways including MTORUP.N4.V1_UP, HOXA9_DN.V1_DN and JAK2_DN.V1_DN were higher enriched in high writer-score group (**Figures S6A, B**; **Tables S4F, G**). Similarly, KEGG pathways related to proliferation and apoptosis such as cell cycle, nucleotide excision repair, and mismatch repair pathways were enriched in high writer-score group (**Figure S6C**; **Table S4H**). Previous research indicated that TP53 and RB1 mutations involved the malignant process of BLCA (51). In our study, we found that the mutation rates of TP53 (50% vs. 46%) and RB1 (18% vs. 16%) were higher in the high writer-score group than in the low writer-score group (**Figure S6D**, **Figure 2C**). The above results explained why the patients in high writer-score group had a poorer prognosis.

### RNA modification patterns predict classical molecular subtypes and therapeutic opportunities in BLCA

The relationships among Cluster, geneCluster, Writer-Score and seven classical molecular subtypes were displayed in **Figures 2A, C**, **Figures S7A, B**. The low writer-score group indicated the luminal subtype (Characterized by luminal differentiation, Ta pathway and urothelial differentiation), while high writer-score group represented the basal subtype (Characterized by immune differentiation, basal differentiation, interferon response, EMT differentiation and keratinization) (**Table S5A**). We also found that patients in the high writer-score group may be more benefit from EGFR targeted therapy and radiotherapy ​, while patients in low writer-score group may be more sensitive to chemotherapies targeting immune-inhibited oncogenic pathways (**Figure 2B**, **Table S5B**). Meanwhile, we successfully verified these results in meta-GEO dataset and E-MTAB-4321 dataset (**Figures S9A–D**; **Table S5C–F**). In summary, RNA modification patterns (Writer-Score) may be used to predict the classical molecular subtypes in BLCA.

### RNA modification patterns predict immune phenotypes and clinical response of ICB in BLCA

**Figure 3:**
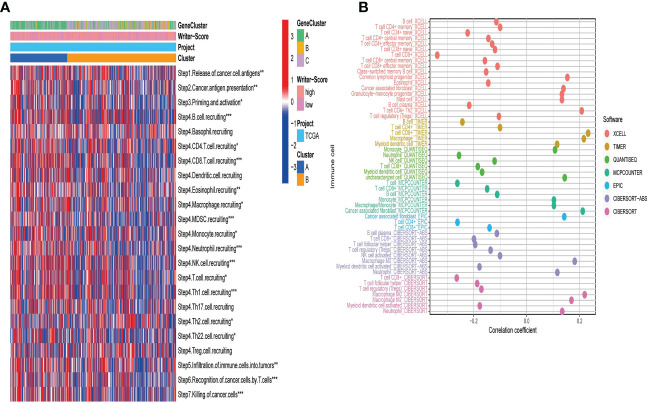
RNA modification patterns (Writer-Score) predict immune phenotypes and clinical response of ICB in the TCGA-BLCA dataset. **(A)** The activities of cancer immunity cycles in different RNA modification patterns. **(B)** The correlation between the Writer-Score and the infiltration levels of TIICs. TCGA, The Cancer Genome Atlas; BLCA, bladder cancer; TIICs, tumor-infiltrating immune cells. *p<0.05, **p<0.01, *** P<0.001.

**Figure 4:**
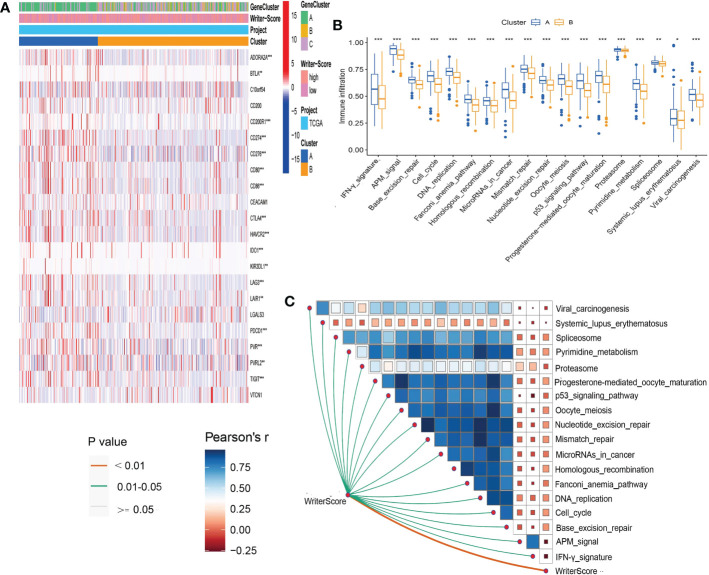
RNA modification patterns (Writer-Score) predict clinical response of ICB in the TCGA-BLCA dataset. **(A)** Differential expression of 22 immune checkpoints in different RNA modification patterns. **(B)** Differential histogram of enrichment scores of positive ICB response-related signatures between RNA modification patterns. **(C)** The correlations between the Writer-Score and enrichment scores of positive ICB response-related signatures. TCGA, The Cancer Genome Atlas; BLCA, bladder cancer; ICB, Immune checkpoint blockade *p<0.05, **p<0.01, *** P<0.001.

A majority of immunomodulators and effector genes of several anticancer TIICs were overexpressed in Cluster A (**Figures S8A, B**; **Tables S6A, B**). The comprehensive performance of immunomodulators can directly determine the activities of cancer immunity cycles. Therefore, we can find that the majority of the steps in the cancer immunity cycle were activated in Cluster A (**Figure 3A**; **Table S7A**). We also Subsequently, we analyzed the relationships between RNA modification patterns and 22 inhibitory immune checkpoints, and found that most of the inhibitory immune checkpoints were overexpressed in ClusterA (**Figure 4A**; **Table S7B**). Meanwhile, the enrichment scores of 18 ICB response-related signatures were enriched in ClusterA (**Figure 4B**).

Then, we explored the correlation between Writer-Score and anticancer immunity of the BLCA. Downregulation of the activity of these cycles will lead to decreased infiltration levels of many anticancer TIICs. Thus, the Writer-Score has negative correlation with most of anticancer TIICs (**Figure 3B**; **Table S8A**). Furthermore, we found that the Writer-Score negatively correlated with the enrichment scores of ICB response-related signatures (**Figure 4C**). Meanwhile, we successfully confirmed these results in meta-GEO dataset and E-MTAB-4321 dataset (**Figures S10**, **S11**; **Table S9A–D**). Moreover, an inflamed phenotype was not only infiltrated by more immune cells, but also more stromal cells (47). The enrichment scores of proliferation signature and four stromal signatures such as EMT1, EMT2, EMT3, and F-TBRS were higher enriched in ClusterA (low writer-score group) (**Figures S8C–F**). Therefore, these findings suggested that ClusterA (low writer-score group writer-score group) may be an inflamed phenotype and be more sensitive to ICB.

### Validating the role of the Writer-Score in predicting immune phenotypes and clinical response to ICB in IMvigor210 dataset

In IMvigor210 dataset, K-M analysis reconfirmed that the patients in high writer-score group have poor prognosis (**Figure S12A**). Patients were classified into several groups according to the PD-L1 expression on immune cells (IC0 subgroup, IC1 subgroup, and IC2 subgroup) and the immune phenotype (deserted phenotype, excluded phenotype and inflamed phenotype) (36, 47). As expected, IC2 subgroup with highest PD-L1 expression on immune cells and inflamed phenotype have lowest Writer-Score (**Figures S12B–D**). In addition, Writer-Score negatively corrected to with the expression level of many inhibitory immune checkpoints and effector genes of anticancer TIICs (**Figures S12E, F**). These results confirmed that the low Writer-Score group may be an inflamed phenotype and be more sensitive to ICB.

Subsequently, we analyzed the relationships between the Writer-Score and ICB response in three distinct immune phenotypes. In the excluded phenotype and inflamed phenotype, the patients in low writer-score group have higher ICB response rate than the patients in high writer-score group (**Figures S12H, I**). Due to the higher ICB response rate, the patients in low writer-score group have better prognosis (**Figures S12K, L**). However, in the deserted phenotype, the patients in high writer-score group have higher ICB response rate and better prognosis than the patients in low writer-score group (**Figures S12G, J**).

### Validating the roles of the Writer-Score in clinical sample dataset

**Figure 5:**
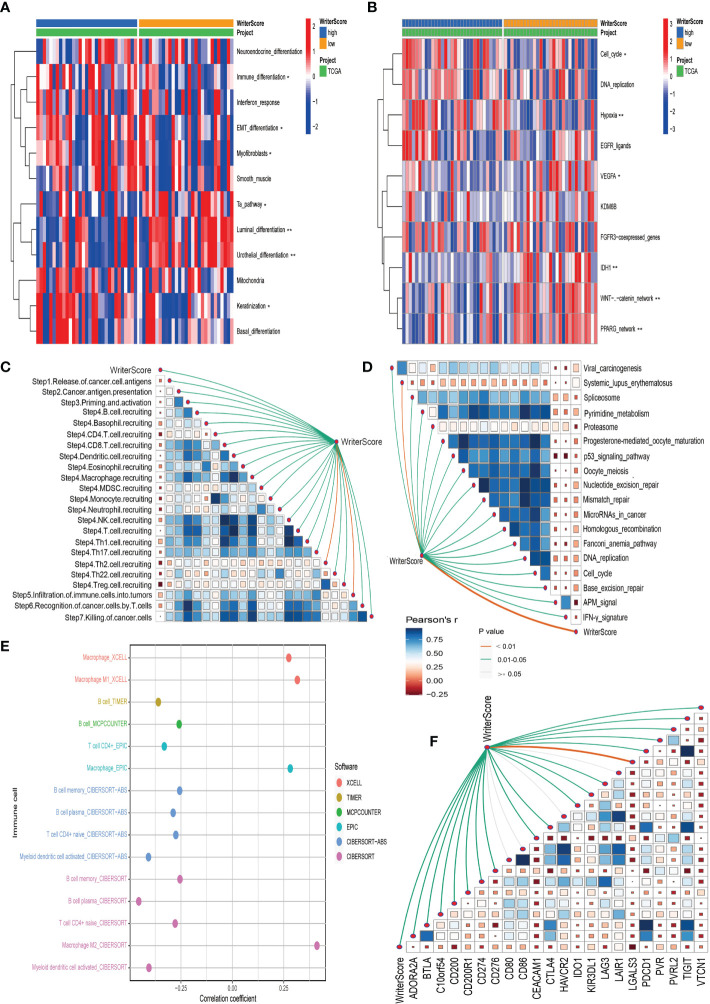
Validating the roles of the Writer-Score in clinical sample dataset. **(A)** The correlations between the Writer-Score and 12 classical molecular subtype-specific signatures. **(B)** The correlations between the Writer-Score and the enrichment scores of several therapeutic signatures, such as EGFR targeted therapy, radiotherapy, chemotherapies targeting immune-inhibited oncogenic pathways. **(C)** The correlations between the Writer-Score and cancer immunity cycles. **(D)** The correlations between the Writer-Score and enrichment scores of positive ICB response-related signatures.**(E)** The correlation between the Writer-Score and the infiltration levels of TIICs. **(F)** The correlations between the Writer-Score and 22 immune checkpoints. TIICs, tumor-infiltrating immune cells. *p<0.05, **p<0.01.

In our own clinical sample dataset, we found that the low writer-score group indicated the luminal subtype, while high writer-score group represented the basal subtype (**Figure 5A**; **Table S5G**). It suggested that the Writer-Score could accurately predict luminal and basal subtypes. Meanwhile, the Writer-Score was negatively related to the activities of many anticancer immunity cycle steps (**Figure 5C**). Subsequently, the Writer-Score was also negatively correlated with the infiltration levels of many anticancer TIICs in seven independent algorithms (**Figure 5E**; **Table S8B**). Next, we found that Writer-Score have negative correlation with the expression level of inhibitory immune checkpoints and enrichment scores of ICB response-related signatures (**Figures 5D, F**). In addition, we also found that patients in the high writer-score group may be more benefit from EGFR targeted therapy and radiotherapy, while patients in low writer-score group may be more sensitive to chemotherapies targeting immune-inhibited oncogenic pathways (**Figure 5B**; **Table S5H**). These finding suggested that the Writer-Score could be used to guide treatments of BLCA.

### Pan-cancer analyses of the Writer-Score

We further assessed the role of the Writer-Score among the cancers and revealed that the Writer-Score was associated with prognosis of many cancers, including brain lower grade glioma (LGG), acute myeloid leukemia (LAML), Mesothelioma (MESO), ovarian serous cystadenocarcinoma (OV), pancreatic adenocarcinoma (PAAD), skin cutaneous melanoma (SKCM) and thyroid carcinoma (THCA) (**Figure S13A**; **Table S10B**). Accumulated evidence indicated that patients with high TMB, MSI and TIS are more sensitive to immunotherapy (35, 47). Here, we found that the Writer-Score was negatively collected with the TMB and MSI in many cancers (**Figures S13B–D**). In addition, we also revealed that the Writer-Score was negatively related to the expression of PD-1, PD-L1, LAG-3 and CTLA-4 in majority of cancers (**Figures S13D–G**). Therefore, the Writer-Score was closely related to many TME characteristics in pan-cancer, suggested that it may be an effective predictor of ICB treatment in cancers.

### The Writer-Score was a valuable predictor to immunotherapy in multiple immunotherapy datasets

**Figure 6:**
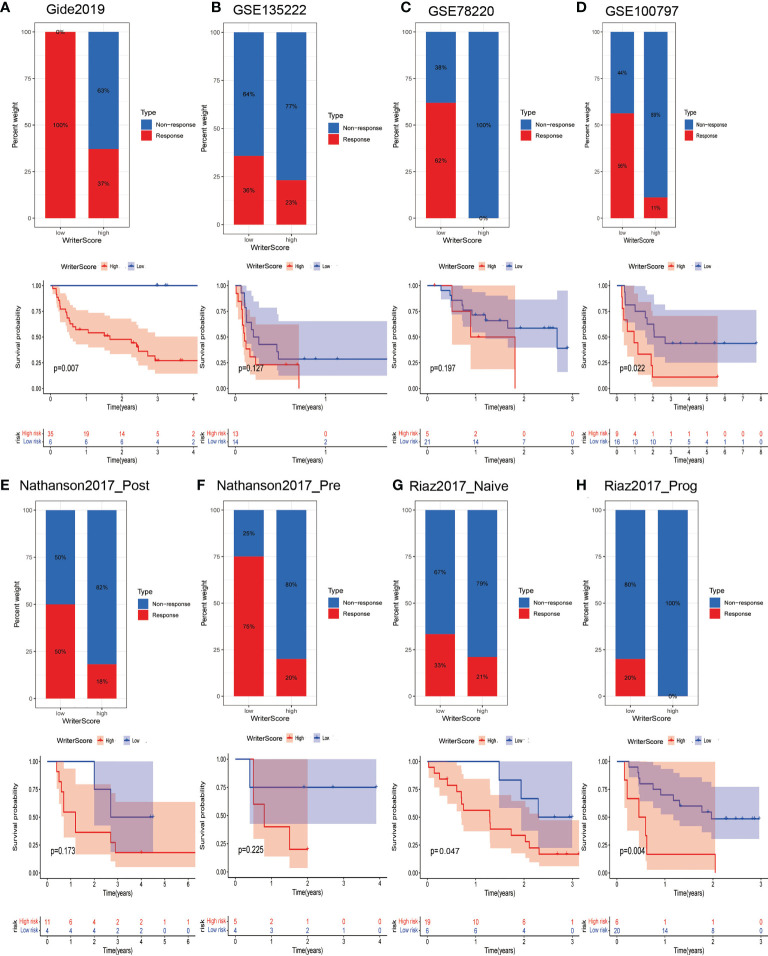
Writer-Score as a valuable predictor to immunotherapy in multiple immunotherapy datasets. **(A–H)** Upper part showed the ICB response rate of patients in low- and high- Writer-Score groups; lower part indicated the prognosis of patients in low- and high- Writer-Score groups. ICB, Immune checkpoint blockade.

We validated the predictive performance of Writer-Score to the immunotherapy in multiple immunotherapy datasets collected from GEO or TIDE database. We found that the Writer-Score was negatively related to inhibitory immune checkpoints in these immunotherapy datasets (**Figures S14**, **S15**). Consistently, the patients in low writer-score group have higher ICB response rate than the patients in high writer-score group. Due to the higher ICB response rate, the patients in low writer-score group have better prognosis (**Figures 6A–H**). These results reconfirmed that the low Writer-Score group may be an inflamed phenotype and be more sensitive to ICB.

## Discussion

Increasing evidence indicate that RNA modification writers play a critical role in tumor immunity. For example, Zhao J et al. (52) revealed that the elevated expression of m6A related genes including ENO1 and PGM1 was positively correlated with infiltration of M2 macrophages and their surface marker CD163, hence affecting the prognosis of BLCA patients. However, previous studies have concentrated on a single type of RNA modification writers, leaving the mutual relationships of multiple RNA modification writer types in BLCA remains unknown. In the present study, we systematically characterized the m1A, m6A, A-to-I and APA RNA editing enzymes at transcriptional and mutation profiles in BLCA. Then, we categorized two distinct clusters, three geneClusters related to RNA modifications based on the expression of 26 RNA modification writers, and constructed a Writer-Score scoring model to quantify all individuals with BLCA. The RNA modification patterns were closely related to prognosis, classical molecular subtypes, tumor immunity and therapeutic strategies in BLCA.

Here, we first systematically assessed the RNA expression profile and somatic mutant profile of 26 RNA modification writers in TCGA-BLCA patients and found that 121 out of 412 patients experienced mutations and that 19 RNA modification writers were abnormal expression in TCGA- BLCA patients. The frequency of mutations ranged from 4% to 1%, and PCF11, METTL3, ZC3H13 had the highest mutations of mutations among the 26 RNA modification writers. The CNV alteration of the 26 RNA modification writers could contribute to their dysregulation expression, and many writers have a significant prognostic value in BLCA. The above results suggested that RNA modification writers play an indispensable role in diagnosis and prognosis of BLCA. We then identified two distinct RNA modification and named them as Cluster A and Cluster B, respectively. Compared with Cluster B, Cluster A was positively corrected with immunological characteristics, such as many immunomodulators, cancer immunity cycles, inhibitory immune checkpoints, many anticancer TIICs and their effector genes. As expect, Cluster B had the poor prognosis in BLCA patients due to the suppression of tumor immunity. In addition, GSVA enrichment analysis indicated that Cluster B was highly enriched in proliferation and apoptosis pathways, such as cell cycle, nucleotide excision repair, and mismatch repair pathways, which explains its poor prognosis.

Furthermore, 1525 differentially expressed mRNA were selected between the two distinct RNA modification patterns. Among them, 355 genes with significant prognostic value were selected to develop a Writer-Score scoring system to quantify the RNA modification patterns of individual BLCA patients. Subsequently, two Writer-Score groups (high- and low writer-score groups) exhibited distinct immunological characteristics. That is, low Writer-Score group appeared to have positively correction with immunological characteristics. K-M analysis revealed that patients in a high Writer-Score group had poor clinical outcome compared with patients in low Writer-Score group. These findings suggested that the Writer-Score was a reliable scoring system for comprehensive clinical assessment of RNA modification patterns in individual BLCA patients, and the low Writer-Score group may be an inflamed phenotype and be more sensitive to ICB. Finally, we reconfirmed these results in multiple immunotherapy datasets and pan-cancer dataset.

More importantly, the low Writer-Score group indicated the luminal subtype, while high Writer-Score group represented the basal subtype. The RNA modification patterns (Writer-Score) also can be used to predict the sensitive to several therapeutic strategies, such as neoadjuvant chemotherapy, EGFR targeted therapy and radiotherapy, chemotherapies targeting immune-inhibited oncogenic pathways. The mutation rate of RB1 was significantly higher in the high Writer-Score group, suggested that the patients in high Writer-Score group (Cluster B) may achieve more benefits from neoadjuvant chemotherapy. In addition, we found that the high Writer-Score group (Cluster B) with hypoxia, cell_cycle, DNA_replication and EGFR_ligands enriched was more sensitive to EGFR targeted therapy and radiotherapy. However, the low Writer-Score group (Cluster A) with several immune-inhibited oncogenic pathways enriched, suggested that targeting these pathways may have advantages for patients in the low Writer-Score group. In summary, the widespread application of the Writer-Score may promote the development of precision medicine in BLCA.

## Conclusions

Our discovery comprehensively analyzed four types of RNA modification writers and develop a reliable Writer-Score scoring model, revealing a novel regulatory mechanism by which they bring some potential implications for identifying molecular subtypes, and guiding therapeutic strategies for BLCA.

## Data availability statement

The datasets presented in this study can be found in online repositories. The names of the repository/repositories and accession number(s) can be found in the article/**Supplementary Material**.

## Ethics statement

This study was approved by the Ethics Committee of Shengjing Hospital of the China Medical University, and informed consent was obtained from all patients. In addition, all methods were performed in accordance with relevant guidelines and regulations. The patients/participants provided their written informed consent to participate in this study.

## Author contributions

YC, YZ, and WW conceived and designed the study. YC, YZ, and WW developed the methodology. YC, YZ, and WW analyzed and interpreted the data. YC, YZ, and WW wrote, reviewed, and/or revised the manuscript. All authors contributed to the article and approved the submitted version.

## Supplementary material

Supplementary Figure 1 Expression of 26 RNA modification writers for all cell types in GSE145137 dataset.

Supplementary Figure 2 Prognostic value of 26 RNA modification writers in TCGA-BLCA dataset. TCGA, The Cancer Genome Atlas; BLCA, bladder cancer.

Supplementary Figure 3 Unsupervised clustering based on 26 RNA modification writers in TCGA-BLCA dataset. **(A-D)** Consensus matrices of the TCGA-BLCA dataset for k = 2-5. **(E-H)** Survival analysis of different RNA modification patterns of the TCGA-BLCA dataset for k = 2-5. **(I, K)** Differential expression of the 26 RNA modification writers between two distinct RNA modification patterns. *P < 0.05; **P < 0.01; ***P < 0.001. **(J)** The differences in KEGG pathways two distinct RNA modification patterns. **(L)** PCA plot of the ClusterA and ClusterB based on 26 RNA modification writers. TCGA, The Cancer Genome Atlas; BLCA, bladder cancer; PCA, Principal Component Analysis; KEGG: Kyoto Encyclopedia of Genes and Genomes.

Supplementary Figure 4 Developing the Writer-score scoring system in TCGA-BLCA dataset. **(A, B)** The volcano plot and venn plot showed the differentially expressed genes (DEGs) between two distinct RNA modification patterns. **(C)** Gene Ontology (GO) analysis of the DEGs. **(D)** Kyoto Encyclopedia of Genes and Genomes (KEGG) analysis of the DEGs. **(E, F)** LASSO regression was applied to establish the Writer-Score scoring system. **(G)** Survival analysis of the high- and low- Writer-Score groups. **(H)** The correlations between Clusters, geneClusters and Writer-Score. **(I)** The distribution diagram of Writer-Score in two distinct clusters. **(J)** The distribution diagram of Writer-Score in three distinct geneClusters. DEGs, differentially expressed genes; LASSO, Least Absolute Shrinkage and Selection Operator.

Supplementary Figure 5 Unsupervised clustering based on DEGs with significant prognostic value. **(A-D)** Consensus matrices of the TCGA-BLCA dataset for k = 2-5. **(E-H)** Survival analysis of different geneClusters of the TCGA-BLCA dataset for k = 2-5. **(I)** Differential expression of DEGs with significant prognostic value between three geneClusters. **(J)** Differential expression of the 26 RNA modification writers between three geneClusters. *P < 0.05; **P < 0.01; ***P < 0.001. **(K)** The forest plot showed the results of multivariable Cox analysis. TCGA, The Cancer Genome Atlas; BLCA, bladder cancer.

Supplementary Figure 6 Functional analyses of RNA modification patterns. **(A)** The differences in hallmark pathways between high- and low- Writer-Score groups. **(B)** The differences in oncogenic pathways between high- and low- Writer-Score groups. **(C)** The differences in KEGG pathways between high- and low- Writer-Score groups. **(D)** The differences in mutational profiles between high- and low- Writer-Score groups. KEGG: Kyoto Encyclopedia of Genes and Genomes.

Supplementary Figure 7
**(A)** The proportions of every subgroup in different molecular subtype systems. **(B)** The distribution of Writer-Score among different subgroups in seven classical molecular subtypes.

Supplementary Figure 8 Correlations between the RNA modification patterns, Writer-Score and immunological characteristics in the TCGA-BLCA cohort. **(A)** The differences in 122 immunomodulators between the RNA modification patterns and high- and low- Writer-Score groups. **(B)** The differences in the expression of effector genes between the RNA modification patterns and high- and low- Writer-Score groups. **(C, D)** The differences in four stromal signature enrichment scores between the RNA modification patterns and high- and low- Writer-Score groups. *P < 0.05; **P < 0.01; ***P < 0.001. **(E, F)** The differences in proliferation signature enrichment score between the RNA modification patterns and high- and low- Writer-Score groups. *P < 0.05; **P < 0.01; ***P < 0.001. TCGA, The Cancer Genome Atlas; BLCA, bladder cancer.

Supplementary Figure 9 The Writer-Score accurately predicted molecular subtypes and therapeutic opportunities in two external validation BLCA cohorts. **(A)** The correlations between the Writer-Score and 12 classical molecular subtype-specific signatures in GEO BLCA meta-cohort (GSE48075, GSE32894). **(B)** The correlations between the Writer-Score and 12 classical molecular subtype-specific signatures in the E-MTAB-4321 dataset. **(C)** The correlations between the Writer-Score and the enrichment scores of several therapeutic signatures, such as EGFR targeted therapy, radiotherapy, chemotherapies targeting immune-inhibited oncogenic pathways in GEO BLCA meta-cohort (GSE48075, GSE32894). **(D)** The correlations between the Writer-Score and the enrichment scores of several therapeutic signatures, such as EGFR targeted therapy, radiotherapy, chemotherapies targeting immune-inhibited oncogenic pathways in the E-MTAB-4321 dataset. GEO, Gene Expression Omnibus; BLCA, bladder cancer.

Supplementary Figure 10 Writer-Score predicts immune phenotypes and ICB response in the GEO BLCA meta-cohort (GSE48075, GSE32894). **(A)** Survival analysis of high- and low- Writer-Score groups. **(B)** The differences in 122 immunomodulators between high- and low- Writer-Score groups. **(C)** The differences in 122 immunomodulators between high- and low- Writer-Score groups. **(D)** The correlations between the Writer-Score and enrichment scores of positive ICB response-related signatures. **(E)** The correlations between the Writer-Score and 22 immune checkpoints. GEO, Gene Expression Omnibus; BLCA, bladder cancer.

Supplementary Figure 11 Writer-Score predicts immune phenotypes and ICB response in the E-MTAB-4321 dataset. **(A)** Survival analysis of high- and low- Writer-Score groups. **(B)** The differences in 122 immunomodulators between high- and low- Writer-Score groups. **(C)** The differences in 122 immunomodulators between high- and low- Writer-Score groups. **(D)** The correlations between the Writer-Score and enrichment scores of positive ICB response-related signatures. ICB, Immune checkpoint blockade.

Supplementary Figure 12 Writer-Score predicts immune phenotypes and clinical response of ICB in the IMvigor210 cohort. **(A)** Survival analysis of high- and low- Writer-Score groups. **(B, C)** The correlation between the Writer-Score and PD-L1 expression on immune cells. **(D)** The differences in the Writer-Score between the three immune phenotypes. **(E)** The correlations between the Writer-Score and 22 immune checkpoints. **(F)** The differences in the expression of effector genes between high- and low- Writer-Score groups. **(G-I)** The proportion of patients responding to ICB in the high- and low- Writer-Score groups in three different immune phenotypes. **(J-L)** Survival analysis of high- and low- Writer-Score groups in three different immune phenotypes. ICB, Immune checkpoint blockade.

Supplementary Figure 13 Pan-cancer analyses of the Writer-Score. **(A)** The prognostic analyses of the Writer-Score across cancers using a univariate Cox regression model. **(B-D)** The correlations between the Writer-Score and TMB, TIS and MSI across cancers. **(E-H)** The correlations between the Writer-Score and four immune checkpoints, PD-1, PD-L1, LAG-3 and CTLA-4. (*P < 0.05; **P < 0.01; ***P < 0.001).

Supplementary Figure 14 Correlations between the Writer-Score and 22 immune checkpoints in four immunotherapy datasets. **(A)** Gide2019 dataset. **(B)** GSE135222 dataset. **(C)** GSE78220 dataset. **(D)** GSE100797 dataset.

Supplementary Figure 15 Correlations between the Writer-Score and 22 immune checkpoints in four immunotherapy datasets. **(A)** Nathanson2017_Post dataset. **(B)** Nathanson2017_ Pre dataset. **(C)** Riaz2017_Naive dataset. **(D)** Riaz2017_ Prog.

Supplementary Table 1 A-P: **(A)** Datasets included in this study; **(B)** Basic information of TCGA-BLCA dataset; **(C)** Basic information of clinical sample dataset; **(D)** Basic information of E-MTAB-4321 dataset; **(E)** Basic information of GSE32894 dataset; **(F)** Basic information of GSE48075 dataset; **(G)** Basic information of IMvigor210 dataset; **(H)** Basic information of TCGA Pan-Cancer dataset; **(I)** Basic information of GSE78220 dataset; **(J)** Basic information of GSE135222 dataset; **(K)** Basic information of Nathanson2017 pre dataset; **(L)** Basic information of Nathanson2017 post dataset; **(M)** Basic information of Gide2019 dataset; **(N)** Basic information of GSE100797 dataset; **(O)** Basic information of Riaz2017_Naive dataset; **(P)** Basic information of Riaz2017_Prog dataset. TCGA, The Cancer Genome Atlas; BLCA, bladder cancer.

Supplementary Table 2 A-L: **(A)** The classification of 25 RNA modification writers; **(B)** The seven classical molecular subtypes in TCGA-BLCA dataset; **(C)** Detailed information of classical molecular subtype-specific signatures; **(D)** 122 immunomodulators; **(E)** The effector genes of tumor associated immune cells; **(F)** The cancer-immunity cycle activity; **(G)** The stromal signatures; **(H)** The pan-cancer T cell inflamed score of TCGA BLCA patients; **(I)** 22 Immune checkpoints; **(J)** Infiltration level of TIICs in pan-cancer using the following algorithms: Cibersort-ABS, Cibersort, MCP-counter, TIMER, quanTIseq, TIP and xCell; **(K)** Detailed information of immunotherapy predicted signatures; **(L)** A set of therapeutic signatures, such as chemotherapies targeting immune-inhibited oncogenic pathways, EGFR targeted therapies and radiotherapies. TCGA, The Cancer Genome Atlas; BLCA, bladder cancer.

Supplementary Table 3 GSVA enrichment analysis showing the activation states of biological pathways in distinct RNA modification patterns. GSVA, Gene set variation analysis.

Supplementary Table 4 A-H: **(A)** 1525 RNA modification-related DEGs between clusterA and cluster; **(B)** GO analysis of 1525 RNA modification-related DEGs; **(C)** KEGG analysis of 1525 RNA modification-related DEGs; **(D)** 355 DEGs with significant prognostic value; **(E)** The coefficients of genes in the Writer-Score scoring system; **(F)** The differences in hallmark pathways between high- and low- Writer-Score groups; **(G)** The differences in oncogenic pathways between high- and low- Writer-Score groups; **(H)** The differences in KEGG pathways between high- and low- Writer-Score groups. DEGs, differentially expressed genes; GO, Gene Ontology; KEGG, Kyoto Encyclopedia of Genes and Genomes.

Supplementary Table 5 A-H: **(A)** The Writer-Score accurately predicted molecular subtypes in TCGA-BLCA dataset; **(B)** The Writer-Score accurately predicted several therapeutic strategies in TCGA-BLCA dataset; **(C)** The Writer-Score accurately predicted molecular subtypes in meta-GEO dataset; **(D)** The Writer-Score accurately predicted several therapeutic strategies in meta-GEO dataset; **(E)** The Writer-Score accurately predicted molecular subtypes in E-MTAB-4321 dataset; **(F)** The Writer-Score accurately predicted several therapeutic strategies in E-MTAB-4321 dataset; **(G)** The Writer-Score accurately predicted molecular subtypes in clinical sample dataset; **(H)** The Writer-Score accurately predicted several therapeutic strategies in in clinical sample dataset. TCGA, The Cancer Genome Atlas; BLCA, bladder cancer; GEO, Gene Expression Omnibus.

Supplementary Table 6 A-B: **(A)** A majority of immunomodulators were overexpressed in Cluster A compared with ClusterB in TCGA-BLCA dataset; **(B)** A majority of effector genes of several anticancer TIICs were overexpressed in Cluster A compared with ClusterB in TCGA-BLCA dataset. TCGA, The Cancer Genome Atlas; BLCA, bladder cancer.

Supplementary Table 7 A-B: **(A)** The majority of the steps in the cancer immunity cycle were activated in Cluster A compared ClusterB; **(B)** The majority of the steps in the inhibitory immune checkpoints were activated in Cluster A compared ClusterB.

Supplementary Table 8 A-B: **(A)** Writer-Score negatively correlated with most of anticancer TIICs in TCGA-BLCA dataset; **(B)** Writer-Score negatively correlated with some anticancer TIICs in clinical sample dataset. TCGA, The Cancer Genome Atlas; BLCA, bladder cancer.

Supplementary Table 9 A-D: **(A)** A majority of immunomodulators were overexpressed in low writer-score group compared with high writer-score group in GEO meta- dataset; **(B)** A majority of effector genes of several anticancer TIICs were overexpressed in low writer-score group compared with high writer-score group in GEO meta- dataset; **(C)** A majority of immunomodulators were overexpressed in low writer-score group compared with high writer-score group in E-MTAB-4321 dataset; **(D)** A majority of effector genes of several anticancer TIICs were overexpressed in low writer-score group compared with high writer-score group in E-MTAB-4321 dataset. GEO, Gene Expression Omnibus; TIICs, tumor-infiltrating immune cells.

Supplementary Table 10 A-B: **(A)** Multivariable Cox analysis revealed that the RNA modification pattern was an independent prognostic factor for BLCA; **(B)** The prognostic analyses of the Writer-Score across cancers using a univariate Cox regression model. BLCA, bladder cancer.
