# Supplementary figures and images for: Four types of adenine-related RNA modification writers -mediated molecular subtypes contribute to predicting clinical outcomes and treatment options in bladder cancer
Source: Front Immunol. 2023 Aug 11;14:1152806. doi: 10.3389/fimmu.2023.1152806 (PMC10450768; doi:10.3389/fimmu.2023.1152806)

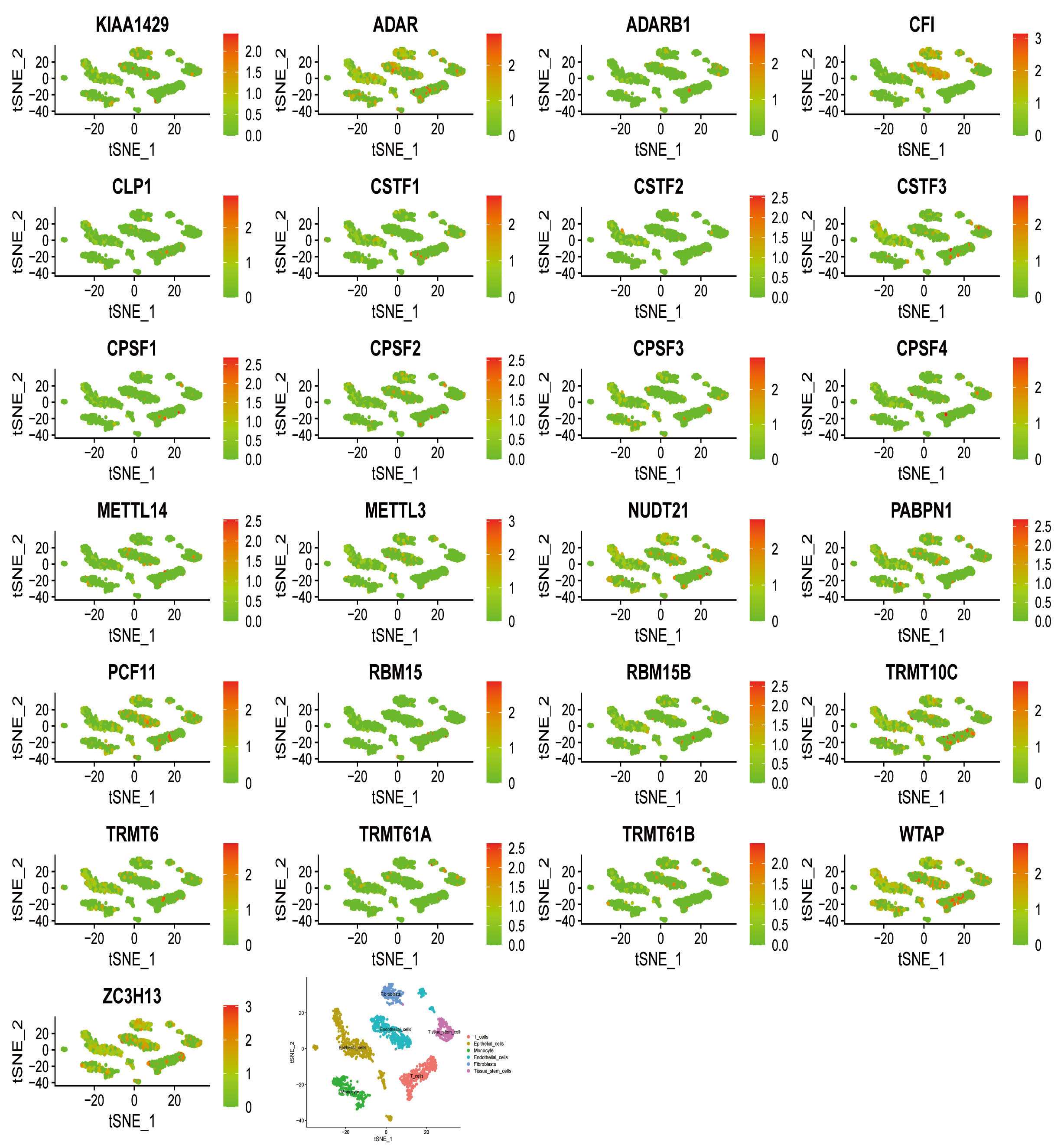

Supplement: Supplementary Figure 1 — Expression of 26 RNA modification writers for all cell types in GSE145137 dataset. [file Image_1.jpeg]

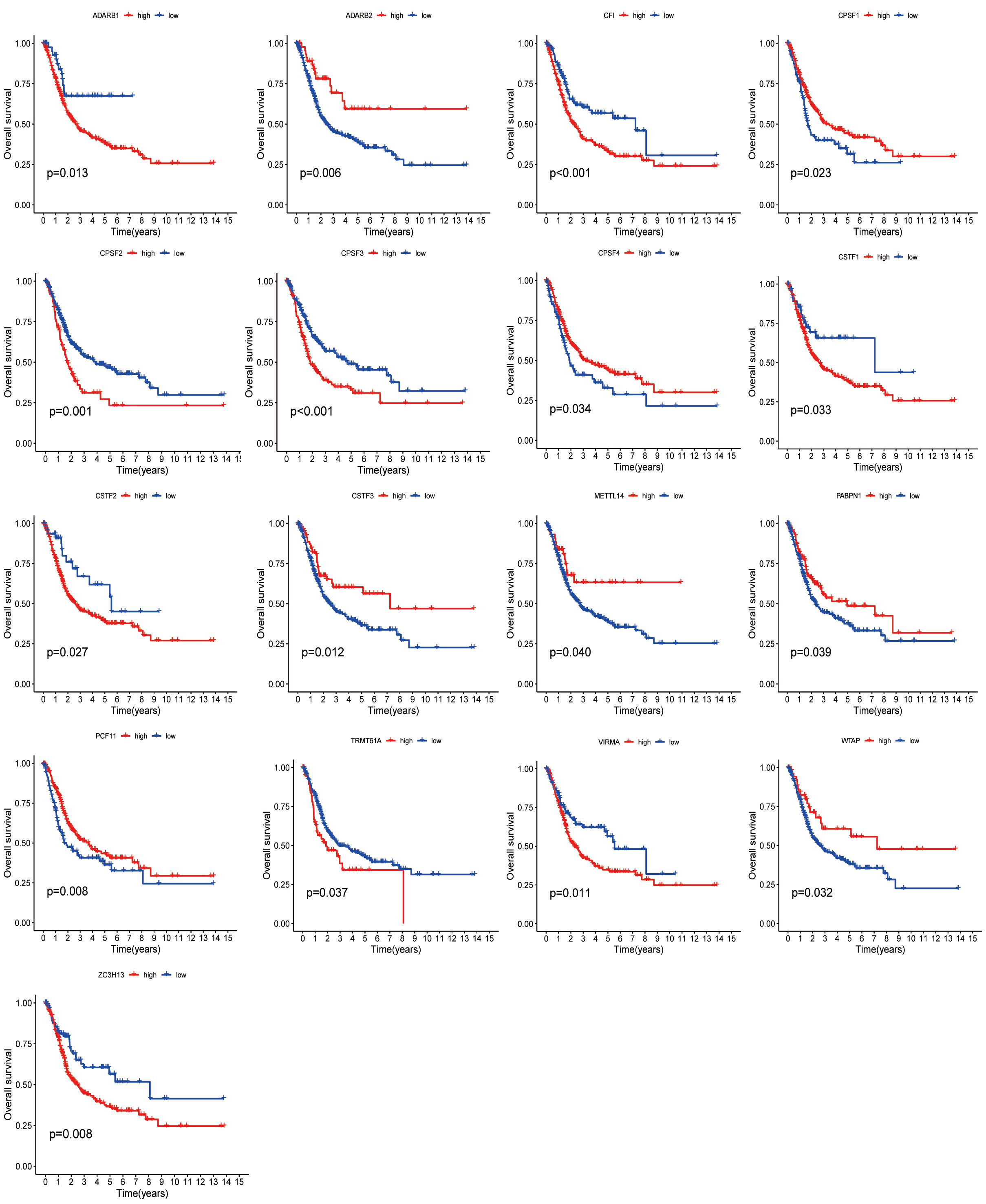

Supplement: Supplementary Figure 2 — Prognostic value of 26 RNA modification writers in TCGA-BLCA dataset. TCGA, The Cancer Genome Atlas; BLCA, bladder cancer. [file Image_2.jpeg]

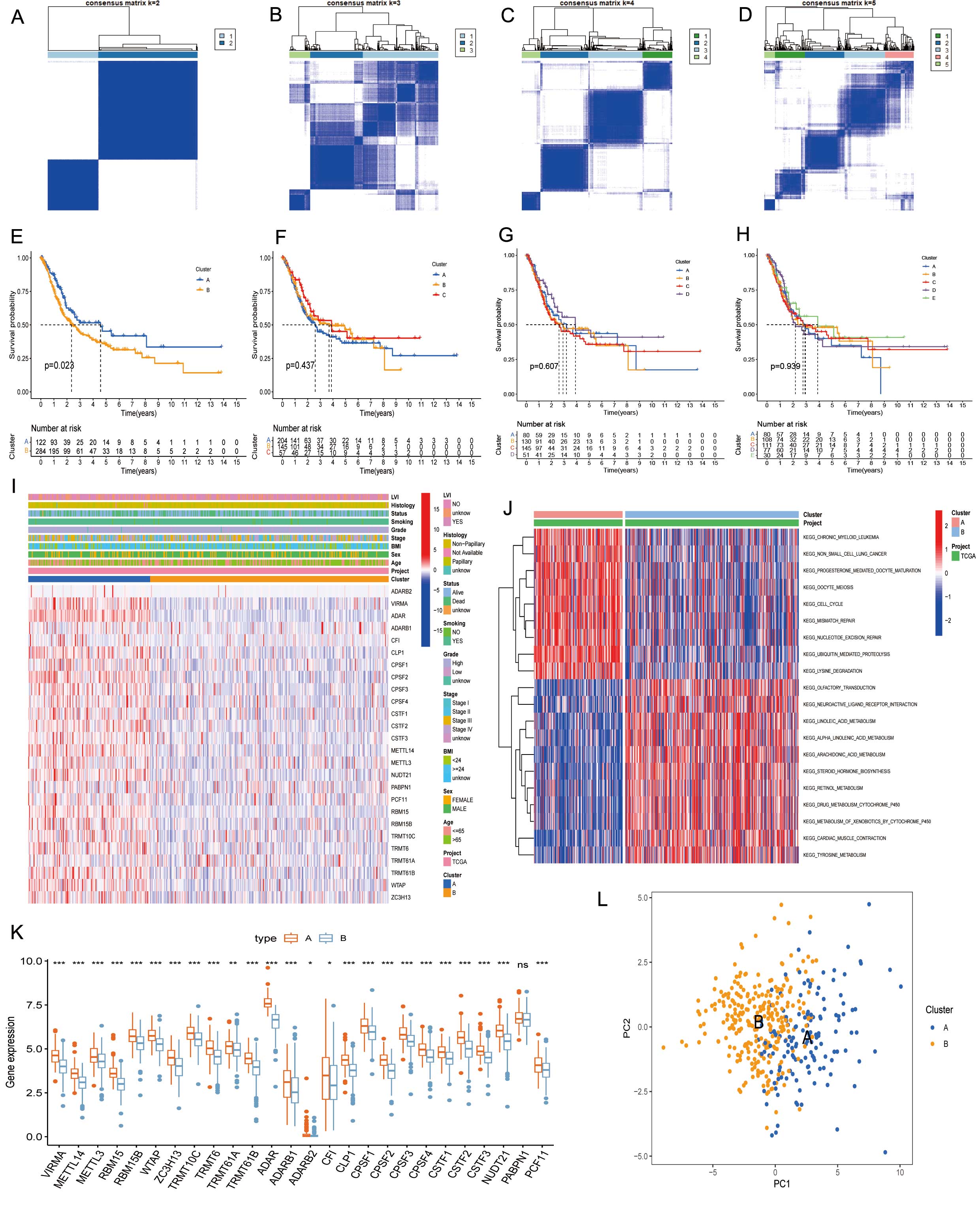

Supplement: Supplementary Figure 3 — Unsupervised clustering based on 26 RNA modification writers in TCGA-BLCA dataset. (A-D) Consensus matrices of the TCGA-BLCA dataset for k = 2-5. (E-H) Survival analysis of different RNA modification patterns of the TCGA-BLCA dataset for k = 2-5. (I, K) Differential expression of the 26 RNA modification writers between two distinct RNA modification patterns. *P < 0.05; **P < 0.01; ***P < 0.001. (J) The differences in KEGG pathways two distinct RNA modification patterns. (L) PCA plot of the ClusterA and ClusterB based on 26 RNA modification writers. TCGA, The Cancer Genome Atlas; BLCA, bladder cancer; PCA, Principal Component Analysis; KEGG: Kyoto Encyclopedia of Genes and Genomes. [file Image_3.jpeg]

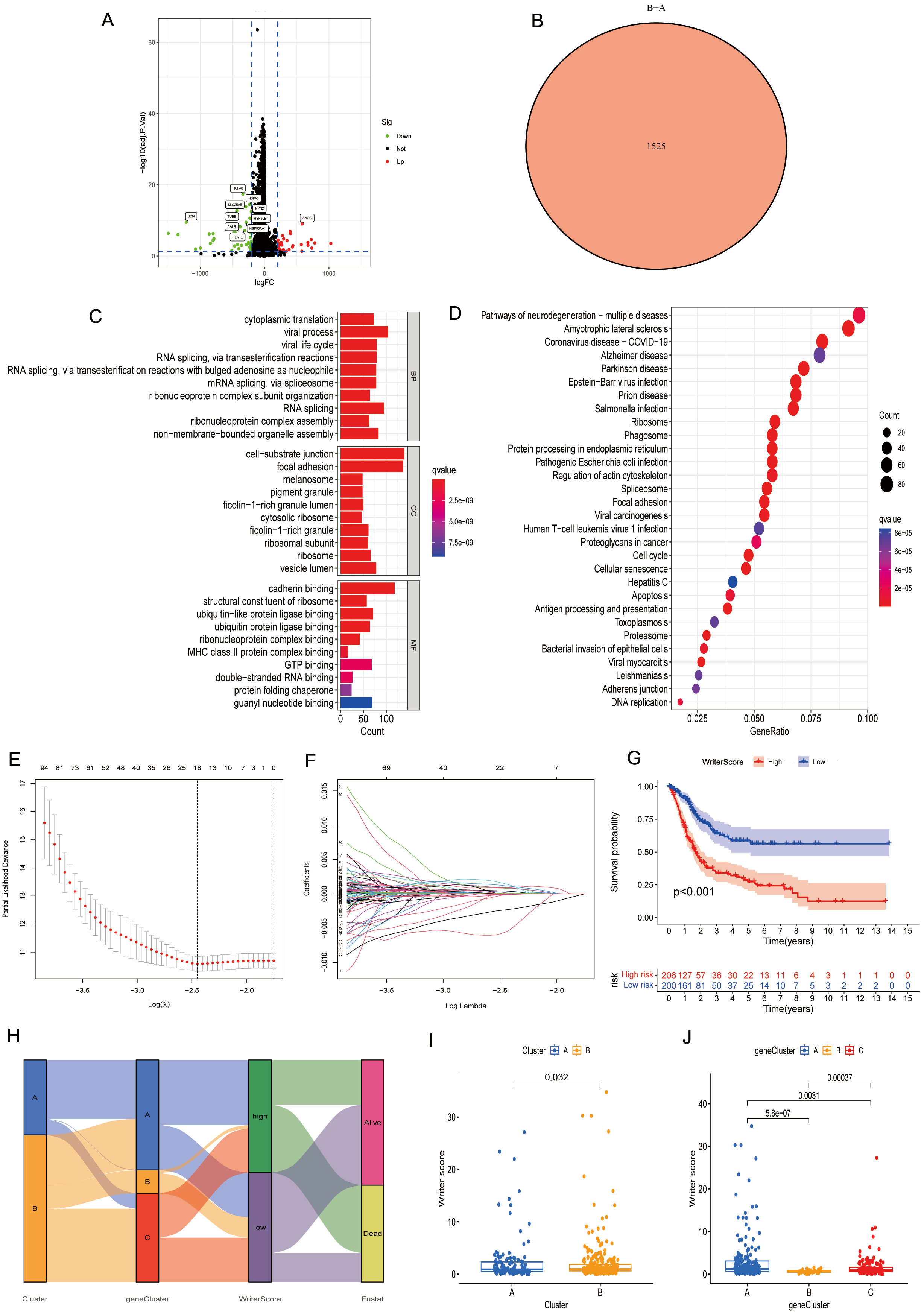

Supplement: Supplementary Figure 4 — Developing the Writer-score scoring system in TCGA-BLCA dataset. (A, B) The volcano plot and venn plot showed the differentially expressed genes (DEGs) between two distinct RNA modification patterns. (C) Gene Ontology (GO) analysis of the DEGs. (D) Kyoto Encyclopedia of Genes and Genomes (KEGG) analysis of the DEGs. (E, F) LASSO regression was applied to establish the Writer-Score scoring system. (G) Survival analysis of the high- and low- Writer-Score groups. (H) The correlations between Clusters, geneClusters and Writer-Score. (I) The distribution diagram of Writer-Score in two distinct clusters. (J) The distribution diagram of Writer-Score in three distinct geneClusters. DEGs, differentially expressed genes; LASSO, Least Absolute Shrinkage and Selection Operator. [file Image_4.jpeg]

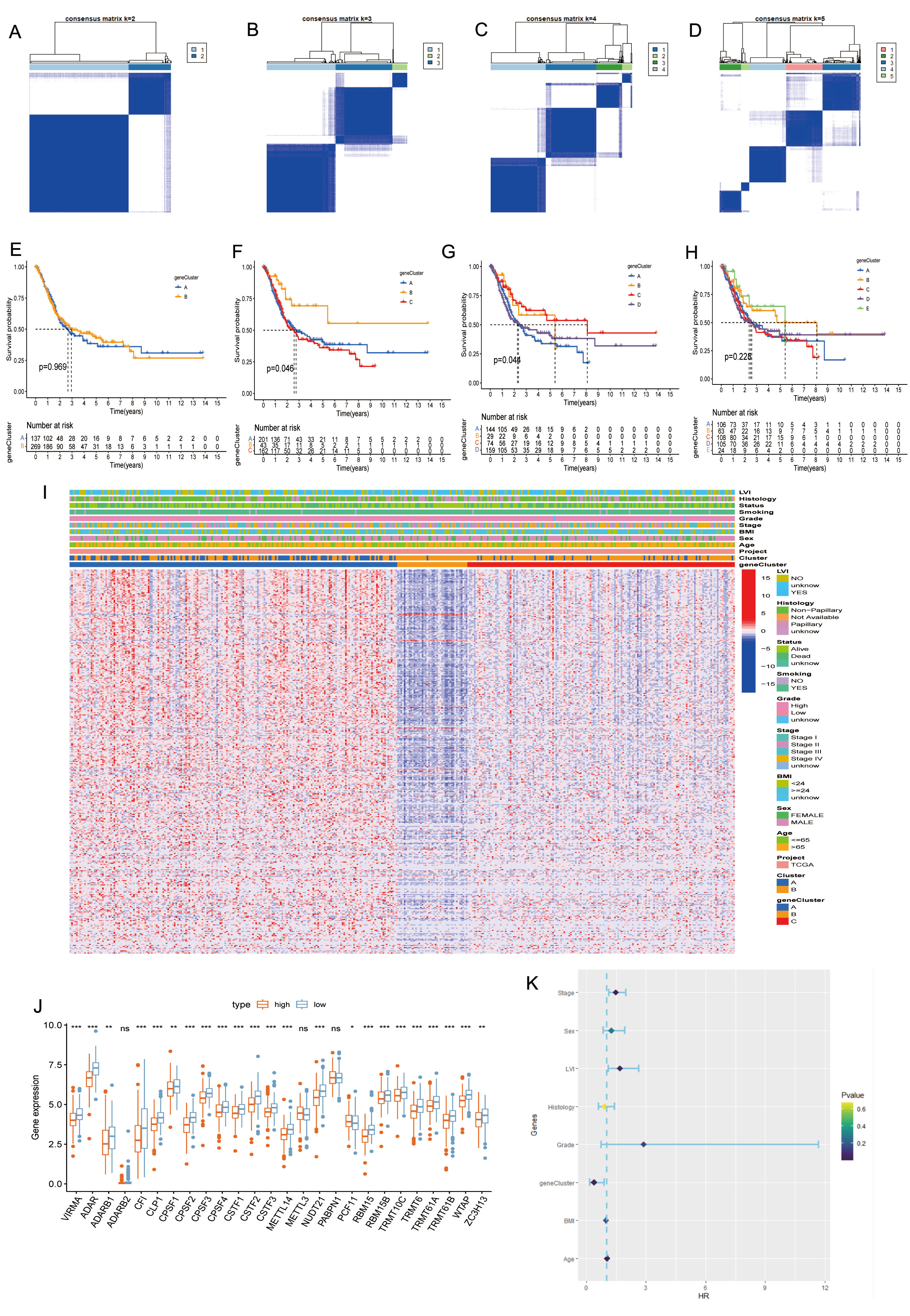

Supplement: Supplementary Figure 5 — Unsupervised clustering based on DEGs with significant prognostic value. (A-D) Consensus matrices of the TCGA-BLCA dataset for k = 2-5. (E-H) Survival analysis of different geneClusters of the TCGA-BLCA dataset for k = 2-5. (I) Differential expression of DEGs with significant prognostic value between three geneClusters. (J) Differential expression of the 26 RNA modification writers between three geneClusters. *P < 0.05; **P < 0.01; ***P < 0.001. (K) The forest plot showed the results of multivariable Cox analysis. TCGA, The Cancer Genome Atlas; BLCA, bladder cancer. [file Image_5.jpeg]

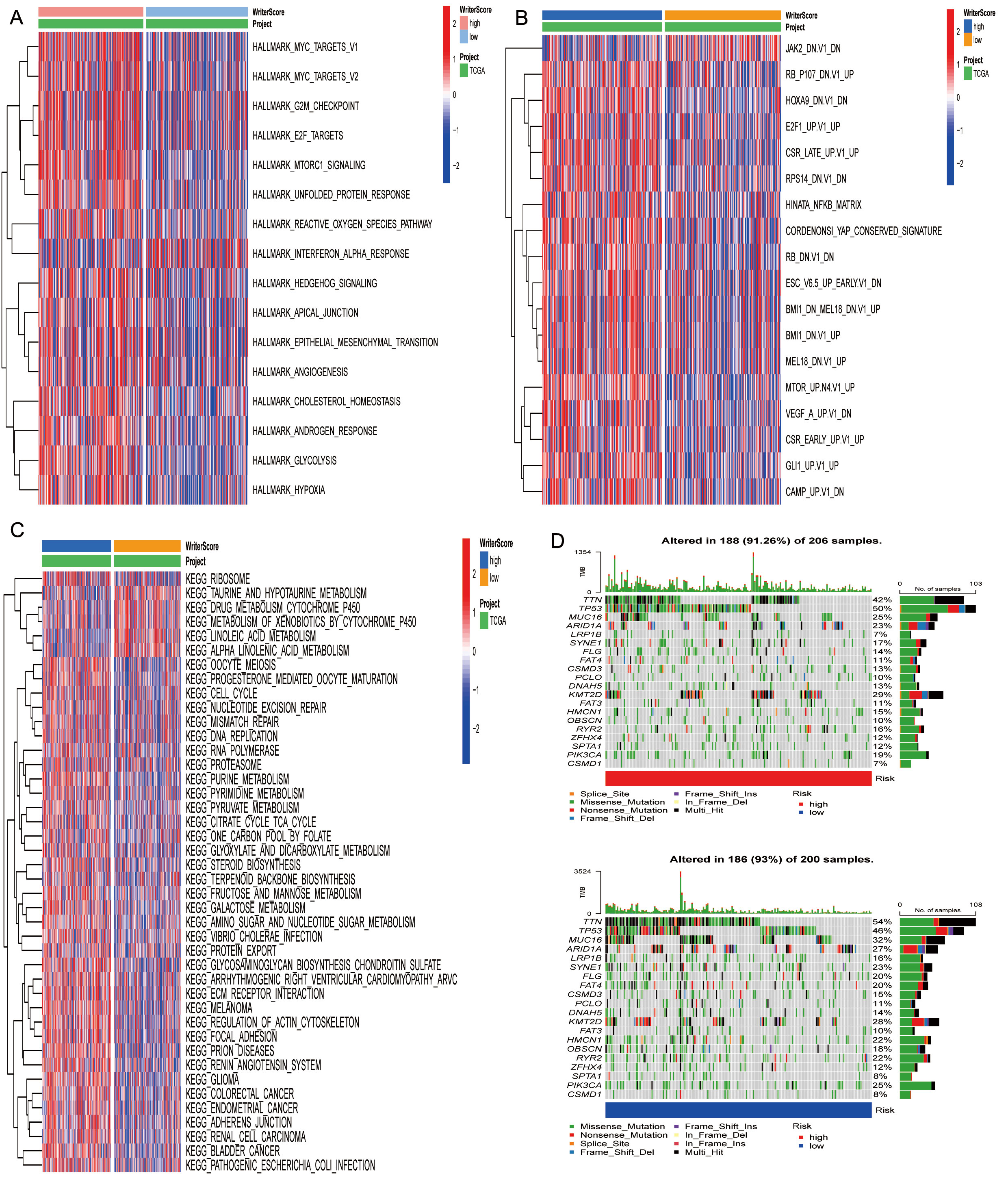

Supplement: Supplementary Figure 6 — Functional analyses of RNA modification patterns. (A) The differences in hallmark pathways between high- and low- Writer-Score groups. (B) The differences in oncogenic pathways between high- and low- Writer-Score groups. (C) The differences in KEGG pathways between high- and low- Writer-Score groups. (D) The differences in mutational profiles between high- and low- Writer-Score groups. KEGG: Kyoto Encyclopedia of Genes and Genomes. [file Image_6.jpeg]

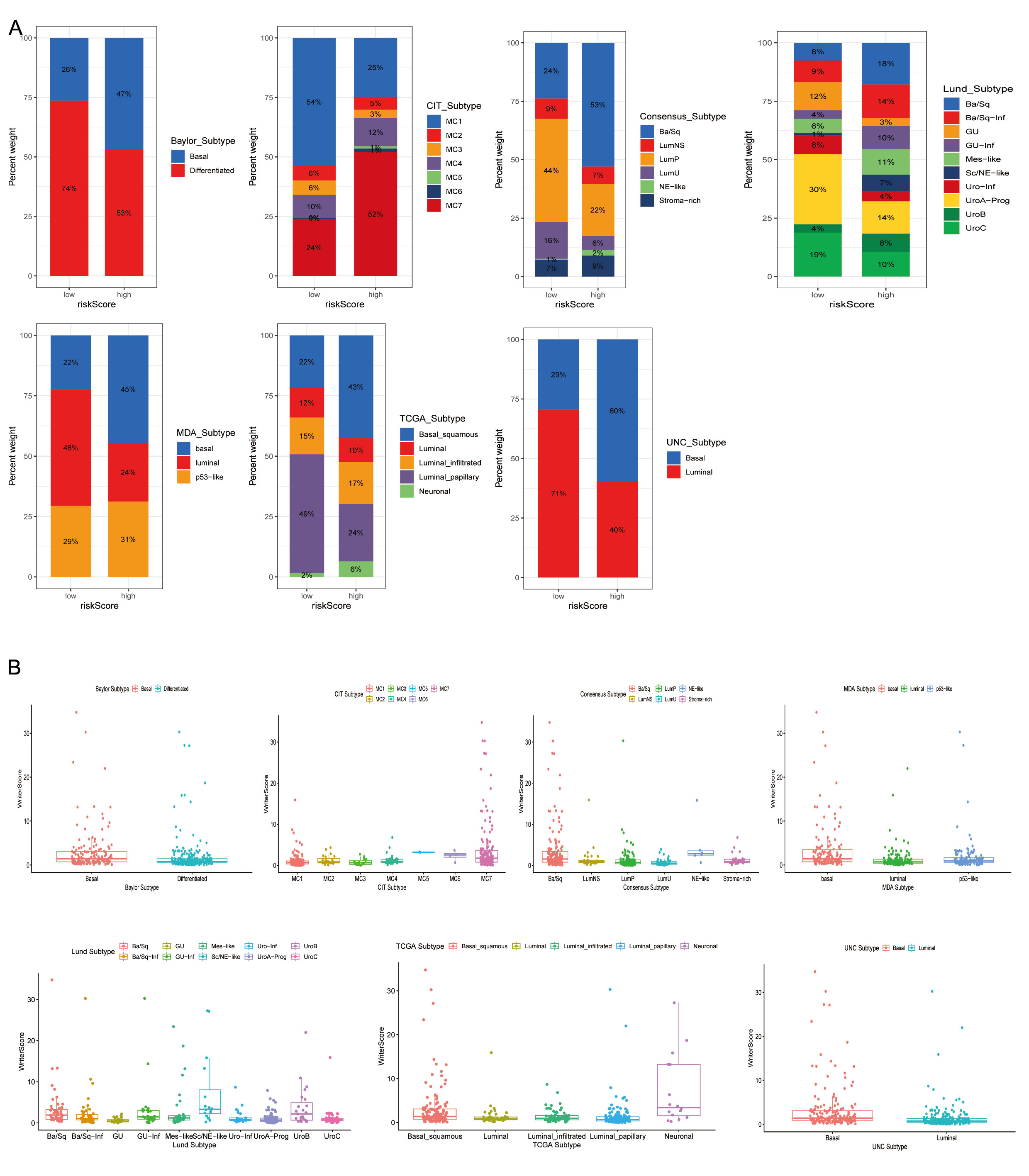

Supplement: Supplementary Figure 7 — (A) The proportions of every subgroup in different molecular subtype systems. (B) The distribution of Writer-Score among different subgroups in seven classical molecular subtypes. [file Image_7.jpeg]

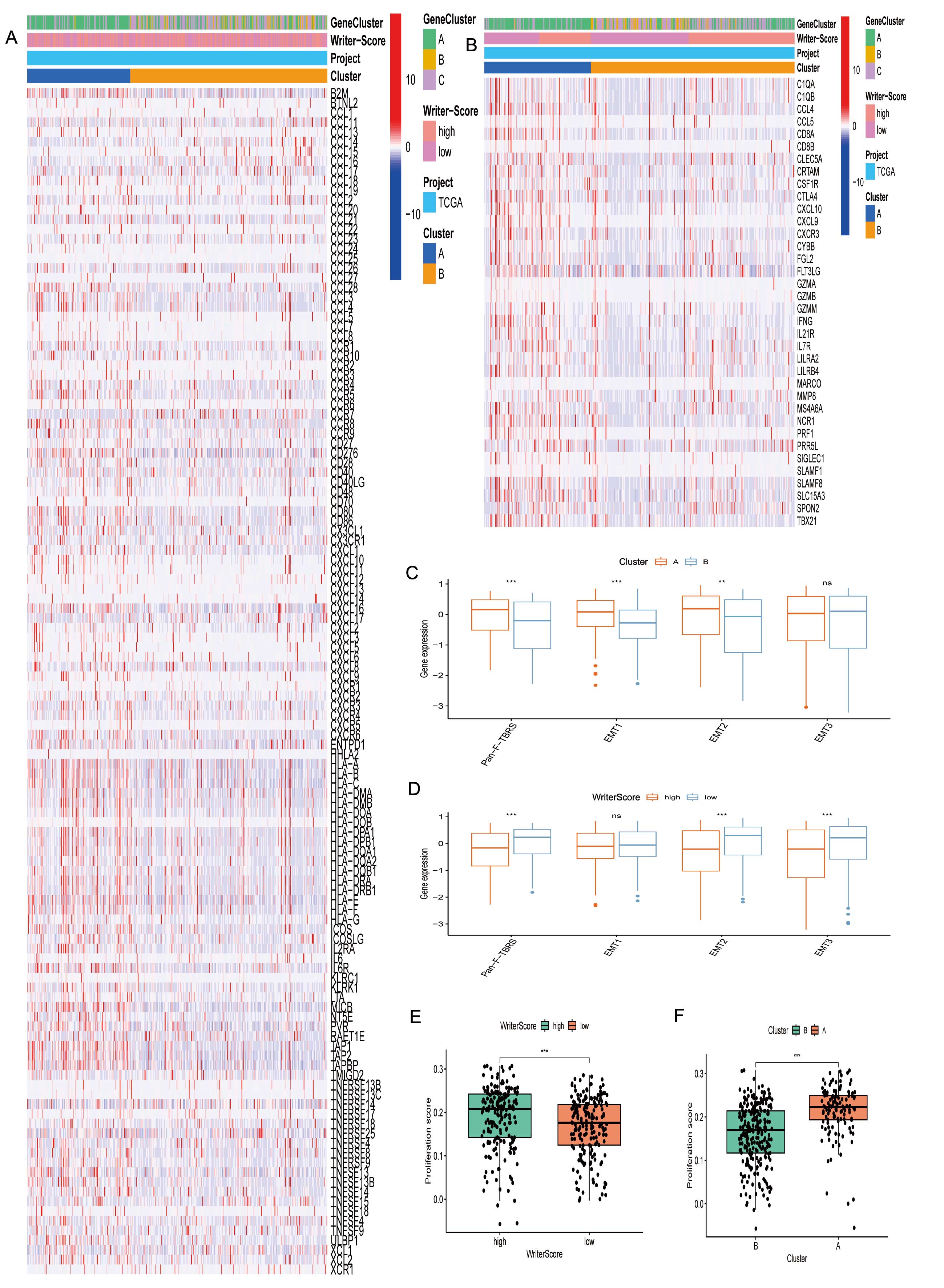

Supplement: Supplementary Figure 8 — Correlations between the RNA modification patterns, Writer-Score and immunological characteristics in the TCGA-BLCA cohort. (A) The differences in 122 immunomodulators between the RNA modification patterns and high- and low- Writer-Score groups. (B) The differences in the expression of effector genes between the RNA modification patterns and high- and low- Writer-Score groups. (C, D) The differences in four stromal signature enrichment scores between the RNA modification patterns and high- and low- Writer-Score groups. *P < 0.05; **P < 0.01; ***P < 0.001. (E, F) The differences in proliferation signature enrichment score between the RNA modification patterns and high- and low- Writer-Score groups. *P < 0.05; **P < 0.01; ***P < 0.001. TCGA, The Cancer Genome Atlas; BLCA, bladder cancer. [file Image_8.jpeg]

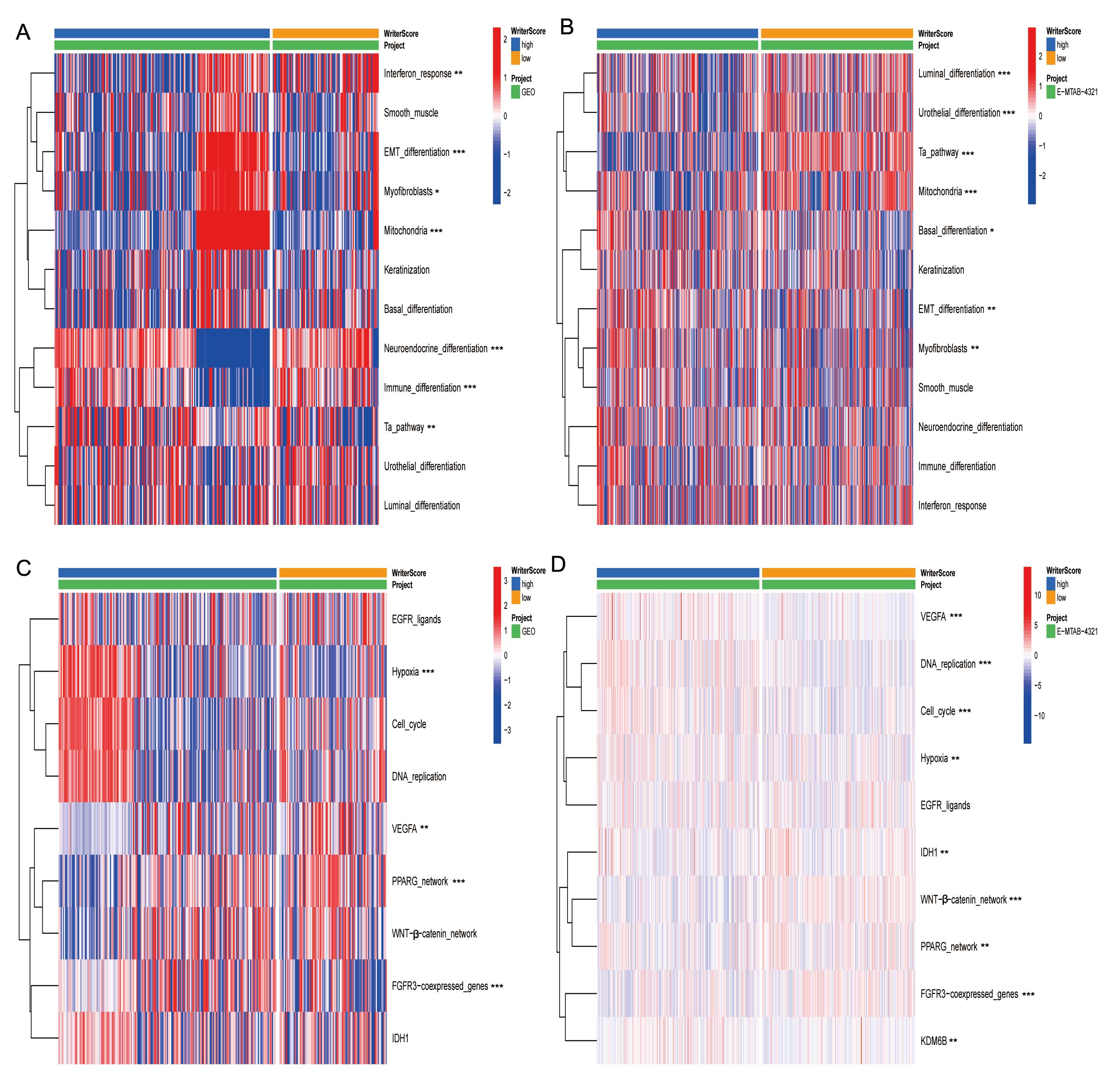

Supplement: Supplementary Figure 9 — The Writer-Score accurately predicted molecular subtypes and therapeutic opportunities in two external validation BLCA cohorts. (A) The correlations between the Writer-Score and 12 classical molecular subtype-specific signatures in GEO BLCA meta-cohort (GSE48075, GSE32894). (B) The correlations between the Writer-Score and 12 classical molecular subtype-specific signatures in the E-MTAB-4321 dataset. (C) The correlations between the Writer-Score and the enrichment scores of several therapeutic signatures, such as EGFR targeted therapy, radiotherapy, chemotherapies targeting immune-inhibited oncogenic pathways in GEO BLCA meta-cohort (GSE48075, GSE32894). (D) The correlations between the Writer-Score and the enrichment scores of several therapeutic signatures, such as EGFR targeted therapy, radiotherapy, chemotherapies targeting immune-inhibited oncogenic pathways in the E-MTAB-4321 dataset. GEO, Gene Expression Omnibus; BLCA, bladder cancer. [file Image_9.jpeg]

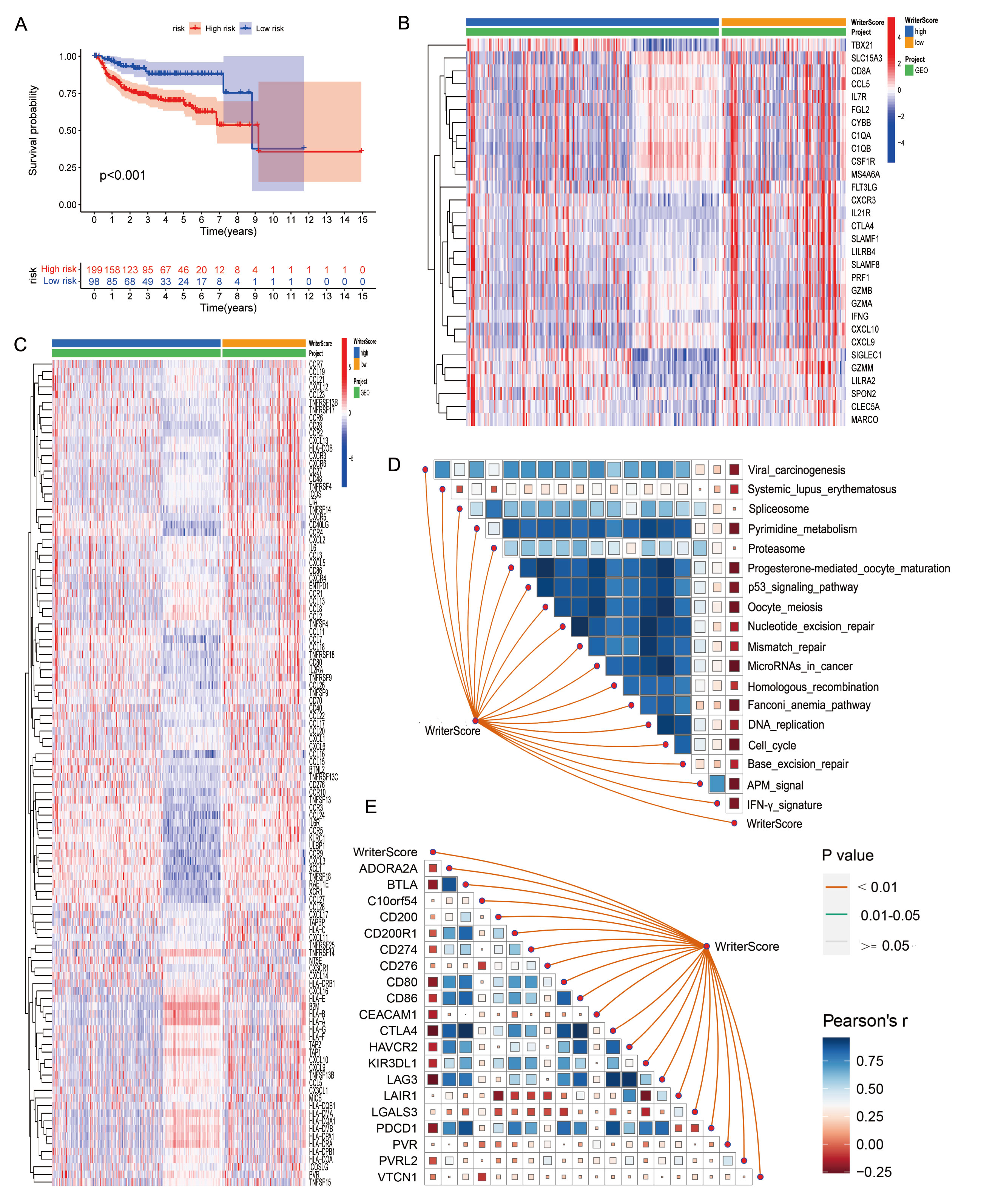

Supplement: Supplementary Figure 10 — Writer-Score predicts immune phenotypes and ICB response in the GEO BLCA meta-cohort (GSE48075, GSE32894). (A) Survival analysis of high- and low- Writer-Score groups. (B) The differences in 122 immunomodulators between high- and low- Writer-Score groups. (C) The differences in 122 immunomodulators between high- and low- Writer-Score groups. (D) The correlations between the Writer-Score and enrichment scores of positive ICB response-related signatures. (E) The correlations between the Writer-Score and 22 immune checkpoints. GEO, Gene Expression Omnibus; BLCA, bladder cancer. [file Image_10.jpeg]

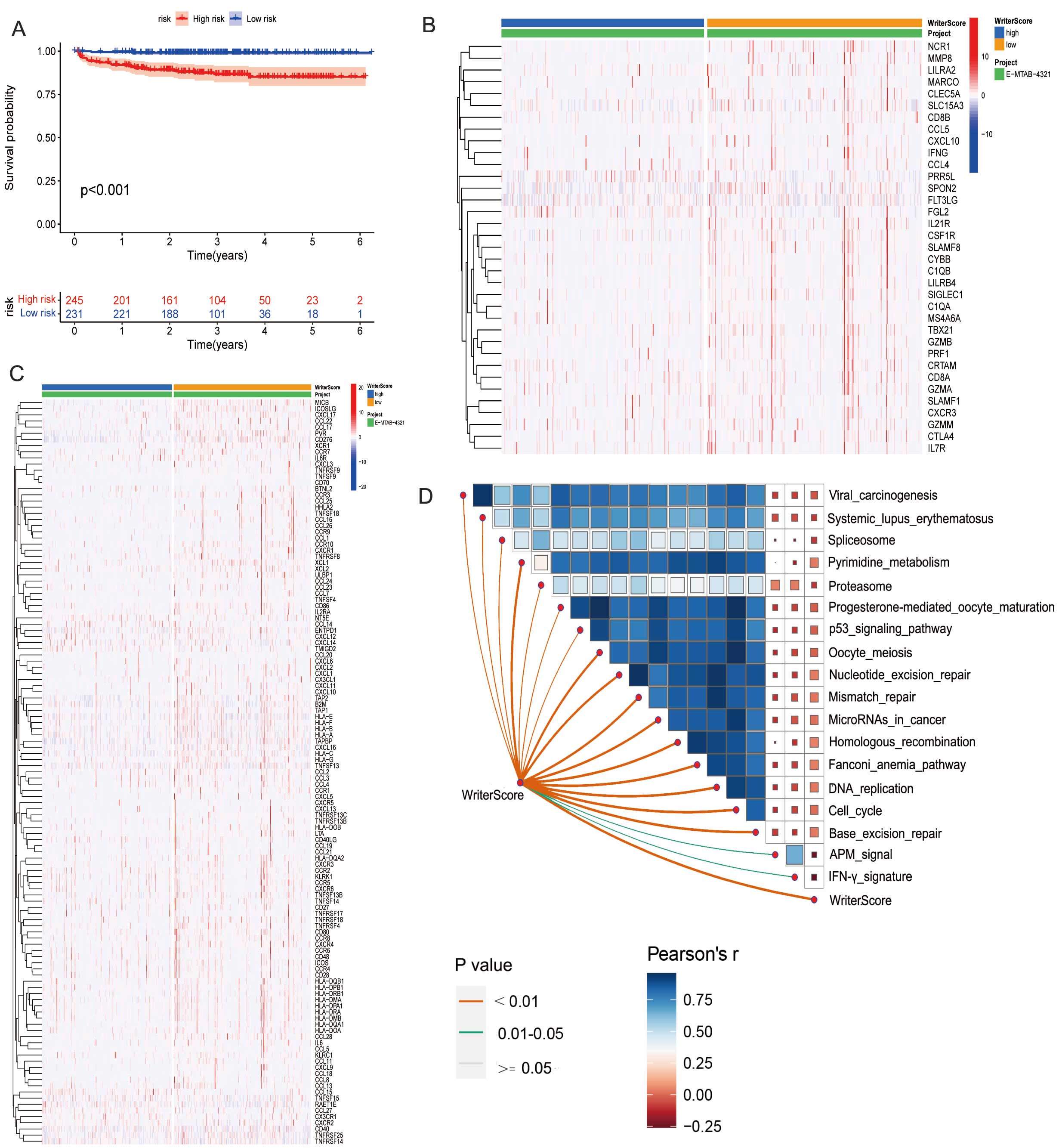

Supplement: Supplementary Figure 11 — Writer-Score predicts immune phenotypes and ICB response in the E-MTAB-4321 dataset. (A) Survival analysis of high- and low- Writer-Score groups. (B) The differences in 122 immunomodulators between high- and low- Writer-Score groups. (C) The differences in 122 immunomodulators between high- and low- Writer-Score groups. (D) The correlations between the Writer-Score and enrichment scores of positive ICB response-related signatures. ICB, Immune checkpoint blockade. [file Image_11.jpeg]

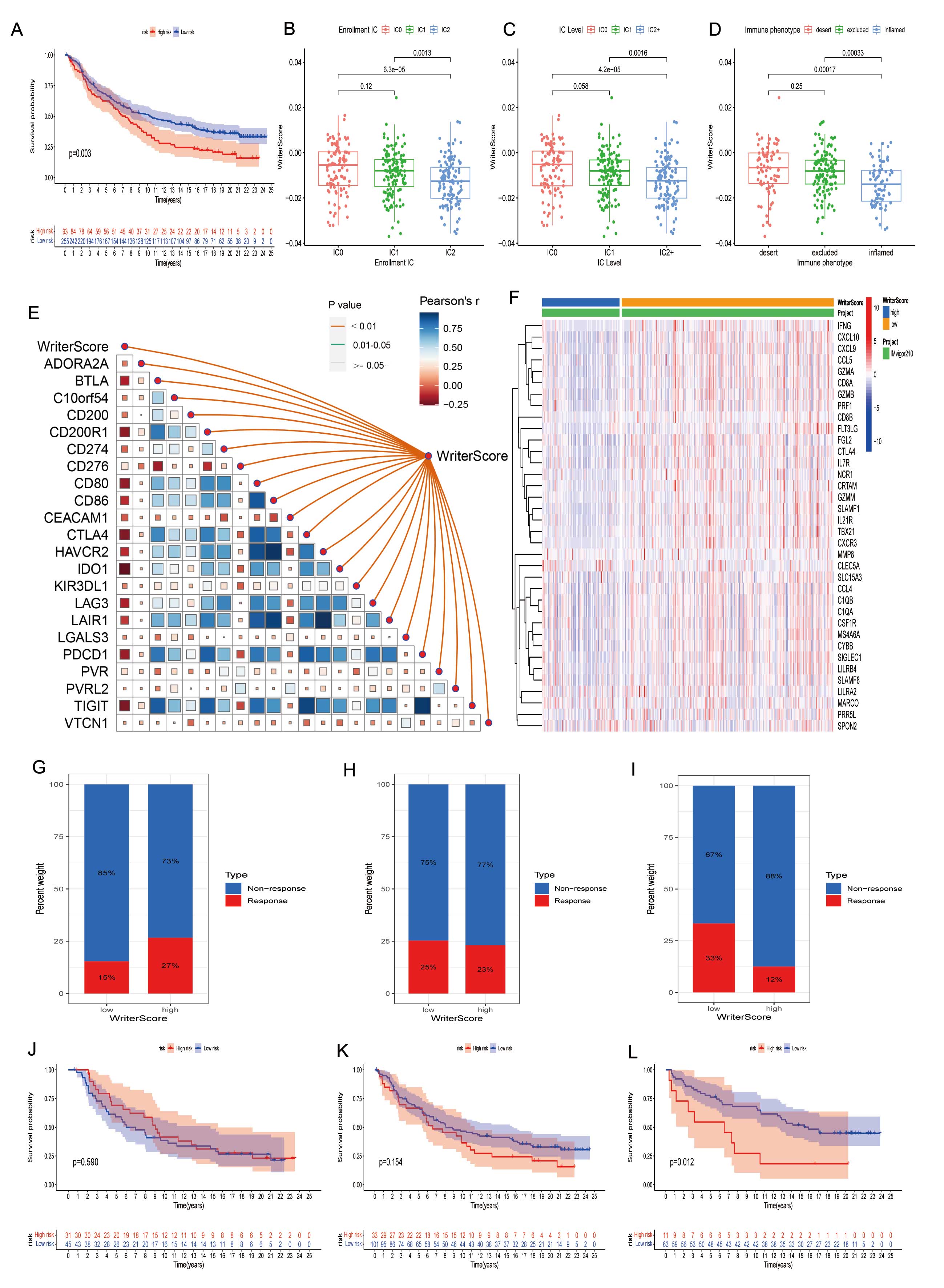

Supplement: Supplementary Figure 12 — Writer-Score predicts immune phenotypes and clinical response of ICB in the IMvigor210 cohort. (A) Survival analysis of high- and low- Writer-Score groups. (B, C) The correlation between the Writer-Score and PD-L1 expression on immune cells. (D) The differences in the Writer-Score between the three immune phenotypes. (E) The correlations between the Writer-Score and 22 immune checkpoints. (F) The differences in the expression of effector genes between high- and low- Writer-Score groups. (G-I) The proportion of patients responding to ICB in the high- and low- Writer-Score groups in three different immune phenotypes. (J-L) Survival analysis of high- and low- Writer-Score groups in three different immune phenotypes. ICB, Immune checkpoint blockade. [file Image_12.jpeg]

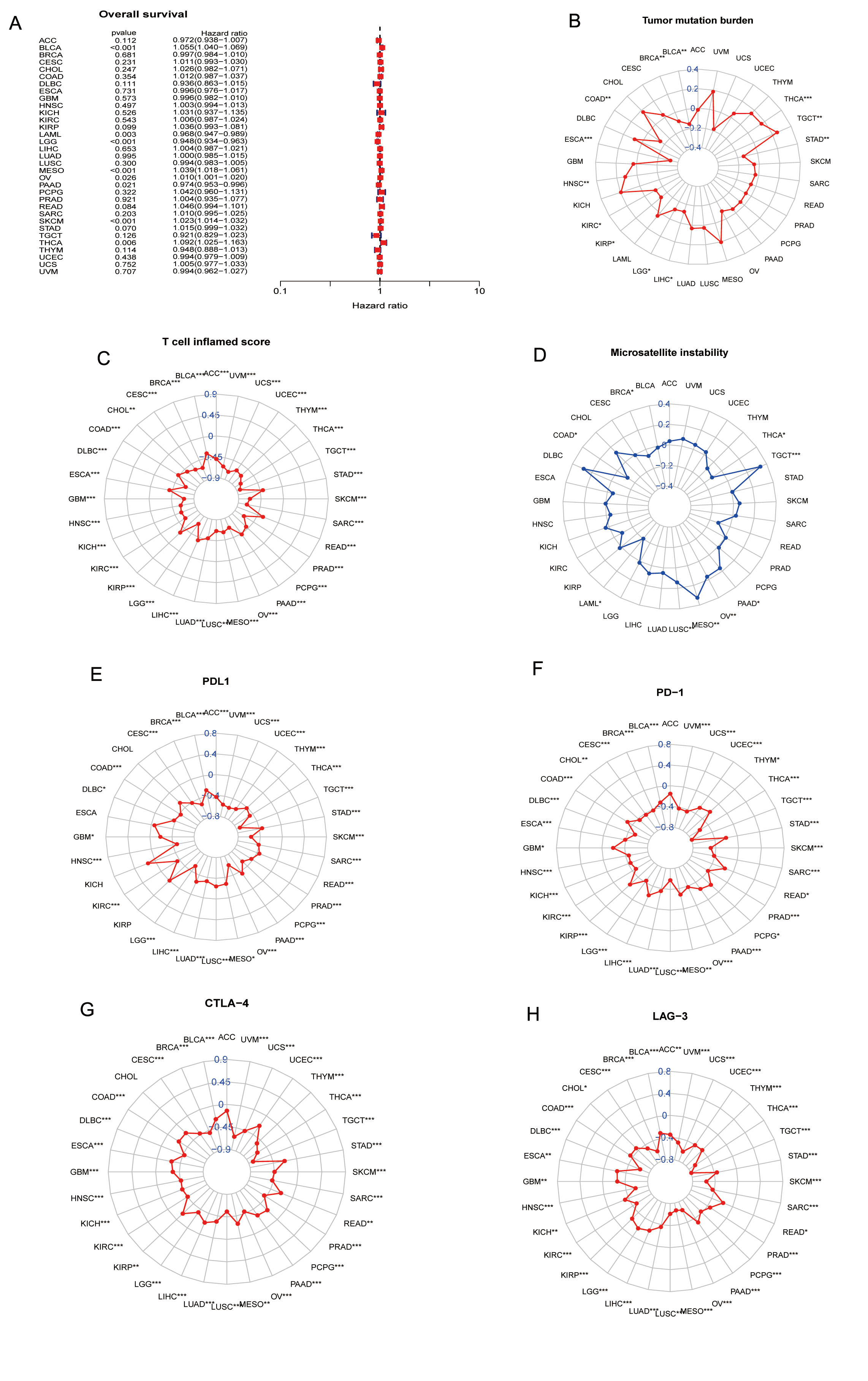

Supplement: Supplementary Figure 13 — Pan-cancer analyses of the Writer-Score. (A) The prognostic analyses of the Writer-Score across cancers using a univariate Cox regression model. (B-D) The correlations between the Writer-Score and TMB, TIS and MSI across cancers. (E-H) The correlations between the Writer-Score and four immune checkpoints, PD-1, PD-L1, LAG-3 and CTLA-4. (*P < 0.05; **P < 0.01; ***P < 0.001). [file Image_13.jpeg]

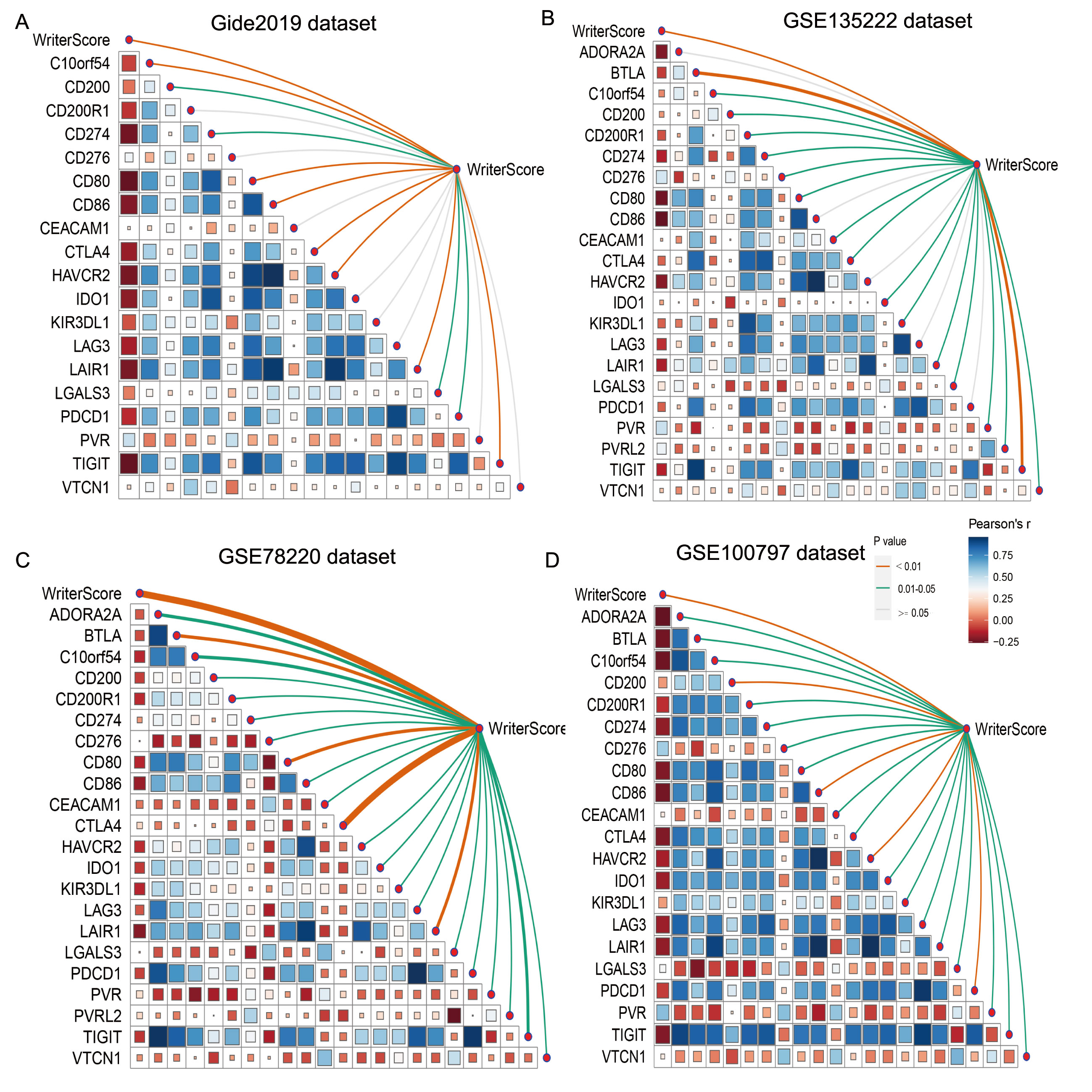

Supplement: Supplementary Figure 14 — Correlations between the Writer-Score and 22 immune checkpoints in four immunotherapy datasets. (A) Gide2019 dataset. (B) GSE135222 dataset. (C) GSE78220 dataset. (D) GSE100797 dataset. [file Image_14.jpeg]

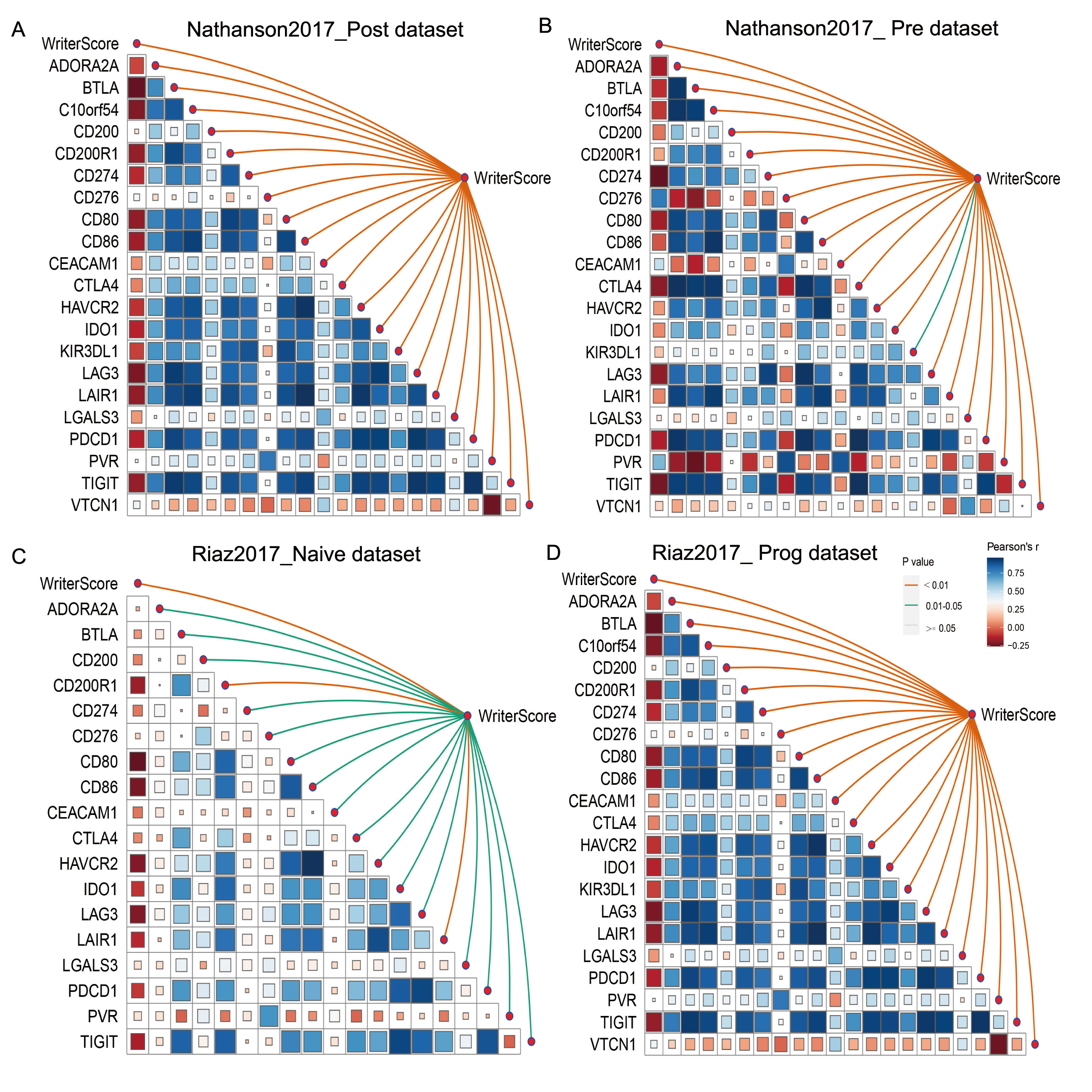

Supplement: Supplementary Figure 15 — Correlations between the Writer-Score and 22 immune checkpoints in four immunotherapy datasets. (A) Nathanson2017_Post dataset. (B) Nathanson2017_ Pre dataset. (C) Riaz2017_Naive dataset. (D) Riaz2017_ Prog. [file Image_15.jpeg]
